# Factors associated with low health-related quality of life in persons with multiple sclerosis: A quantile-based segmentation approach

**DOI:** 10.1371/journal.pone.0312486

**Published:** 2024-11-21

**Authors:** Stefania Iaquinto, Benjamin Victor Ineichen, Anke Salmen, Jens Kuhle, Pascal Benkert, Lisa Hofer, Pasquale Calabrese, Christian P. Kamm, Patrick Roth, Chiara Zecca, Sabin Ammann, Caroline Pot, Viktor von Wyl, for the Swiss Multiple Sclerosis Registry

**Affiliations:** 1 Epidemiology, Biostatistics and Prevention Institute, University of Zurich, Zurich, Switzerland; 2 Center for Reproducible Science (CRS), University of Zurich, Zurich, Switzerland; 3 Department of Neurology, St. Josef-Hospital Bochum, Ruhr-University Bochum, Bochum, Germany; 4 Department of Neurology, University Hospital Basel and University of Basel, Basel, Switzerland; 5 Neurology Clinic and Polyclinic, Departments of Head, Spine and Neuromedicine, MS Center and Research Center for Clinical Neuroimmunology and Neuroscience Basel (RC2NB), Clinical Research and Biomedical Engineering, University Hospital Basel and University of Basel, Basel, Switzerland; 6 Clinical Trial Unit, Department of Clinical Research, University Hospital Basel and University of Basel, Basel, Switzerland; 7 Division of Cognitive and Molecular Neuroscience, Neuropsychology and Behavioral Neurology Unit, University of Basel, Basel, Switzerland; 8 Department of Neurology, Inselspital, Bern University Hospital, University of Bern, Bern, Switzerland; 9 Neurocentre, Lucerne Cantonal Hospital, Lucerne, Switzerland; 10 Department of Neurology and Brain Tumor Center, University Hospital Zurich and University of Zurich, Zurich, Switzerland; 11 Department of Neurology, Neurocenter of Southern Switzerland, Ospedale Regionale di Lugano, EOC, Lugano, Switzerland; 12 Faculty of Biomedical Sciences, Università della Svizzera Italiana, Lugano, Switzerland; 13 Service of Neurology, Department of Clinical Neurosciences, Lausanne University Hospital (CHUV) and University of Lausanne, Lausanne, Switzerland; 14 Institute for Implementation Science in Health Care, University of Zurich, Zurich, Switzerland; Fondazione Don Carlo Gnocchi, ITALY

## Abstract

**Background:**

Improving health-related quality of life (HRQoL) is an important disease management goal in persons with Multiple Sclerosis (PwMS). HRQoL decreases with increasing age and prolonged disease duration; other factors remain less understood.

**Objective:**

To identify associations of multiple sclerosis (MS) disease characteristics and symptom burden with low HRQoL.

**Methods:**

Using the Swiss MS Registry, we applied quantile regression adjusted for age and MS disease duration to determine 25^th^ (low HRQoL) and 75^th^ (high HRQoL) percentiles of the EuroQol-5-Dimension (EQ-5D) distribution for PwMS. We compared PwMS across HRQoL groups by analyzing differences in sociodemographics, symptom burden, MS risk factors, gait impairment, and the MS Severity Score (MSSS), all measured at the same time as HRQoL. The analyses included descriptive methods, multivariable multinomial regression, and simultaneous quantile regression as a sensitivity analysis.

**Results:**

We included 1697 PwMS with median age and time-to-diagnosis of 49 and 9 years. Multivariable regression revealed low HRQoL to be associated with receiving invalidity insurance benefits, reporting depression, muscle weakness, memory problems, pain, and severe gait impairment. The analysis for individuals with available MSSS (n = 937) showed an increasing probability of low HRQoL with higher MSSS.

**Conclusion:**

Our segmentation method identified symptom burden and MS severity as factors associated with low HRQoL. Pharmacological and non-pharmacological MS symptom management, especially for depression, fatigue, pain, and muscle weakness, may warrant increased attention to preserve or improve HRQoL.

## Introduction

Multiple Sclerosis (MS) is a chronic neurodegenerative autoimmune disease that affects approximately 3 million people worldwide [1], with a typical disease onset between 20 and 40 years [2]. Persons with MS (PwMS) may suffer from highly individualized and very diverse symptom combinations, often including visual problems, paresthesia, neuropsychiatric disturbances, gait impairment, or fatigue. This considerable symptom burden, as well as frequent daily-life impairments and concomitant diseases (comorbidities), diminish the health-related quality of life (HRQoL), which is often lower in PwMS when compared to the general population [3–6]. Studies have identified specific MS symptoms, including fatigue, depression, gait impairment, or spasticity, as having particularly detrimental effects on HRQoL [4, 5, 7]. Furthermore, a recent investigation into predictors of HRQoL trajectories in PwMS also noted baseline associations of older age, physical impairments, or fatigue with a worse HRQoL trajectory [6].

However, the factors shaping and predicting long-term HRQoL are still poorly understood. These knowledge gaps concerning drivers of HRQoL in PwMS are consequential and hinder a more personalized, HRQoL-oriented management of MS and its symptoms. Previous studies have investigated HRQoL influences using, for example, median regression or trajectory analysis using latent growth models. These studies already offered important insights, but also faced a multitude of analytical challenges for identifying individual-level drivers of low HRQoL. For example, although simple, well validated, and frequently employed, generic HRQoL instruments such as the EuroQol-5-Dimension (EQ-5D) scale only cover a small subset of MS-related health aspects, and they are often not very responsive to short-term changes in symptom severity or disease exacerbation [8]. Also, the EQ-5D and similar non-disease-specific scales are usually normed for the general population, which limits the clinical usefulness for persons with chronic diseases. Rather, such HRQoL measures should be compared against and interpreted in the context of measurement distributions from persons with similar disease characteristics. Finally, PwMS live with their disease for decades, which leads to complex interactions between HRQoL and natural aging and disease progression that are difficult to disentangle.

To overcome these analytical challenges and to offer a novel methodological approach, we used a cross-sectional, observational study design to explore a quantile regression-based segmentation approach that aims to robustly identify individuals with low HRQoL within the PwMS population and after adjustment for age- and disease duration dependent effects on HRQoL. Disentangling age-related from disease-related health changes is complex because they are interwoven and can lead to problems of multicollinearity in statistical analyses. Over long time-horizons, HRQoL and health changes can occur both due to biological aging and disease progression, such as increasing impairment of ambulation.

By applying this segmentation analysis to the database of the Swiss Multiple Sclerosis Registry (SMSR), we sought to compare PwMS with low or high HRQoL after age and disease duration adjustment, and to identify independent disease- and symptom-related characteristics associations with low HRQoL. Our proposed analytic approach mitigates the problem of multicollinearity and uses the population of PwMS as a reference to identify persons with low or high HRQoL.

## Materials and methods

### Study population

This study includes SMSR study participants. The SMSR has been described extensively elsewhere [9]. The SMSR, a nationwide, longitudinal study of PwMS in Switzerland, offers two participation modes: a one-time survey with basic information for epidemiological studies, and longitudinal participation, including an extended baseline survey and invitations to digital or paper-based semi-annual follow-ups. Active since 2016, the SMSR collected one-time surveys from 2800 PwMS living or receiving care in Switzerland, with 2200 also contributing to the longitudinal study. The SMSR closely collaborates and is advertised by the Swiss Multiple Sclerosis Society and numerous neurological centers in Switzerland. PwMS aged 18 years and older can self-enroll the SMSR online or request paper-based questionnaires from the study center at the University of Zurich. Before the first questionnaire, prospective participants complete an informed consent, including opt-in agreements for medical record release or data linkage with the Swiss Multiple Sclerosis Cohort Study (SMSC) [10]. The SMSR relies on self-reports and mainly collects information on health-related quality of life and lived experience with MS. The SMSC covers high-quality clinical data and disability assessments, MRI, and biomarkers. The SMSC is conducted on-site at eight large MS centers and integrated into routine neurological care. As of December 31, 2022, approximately 1600 participants with a median follow-up period of 6.2 years have been enrolled. About 250 individuals also contribute to the SMSR and have signed informed consents for joint data linkage.

### Databases

This study primarily used data from the SMSR database, including HRQoL and other self-reported measures such as sociodemographic information, MS disease characteristics, MS treatments, MS symptoms, disability status, chronic comorbidities, and familial MS risk. Additionally, the study utilized Expanded Disability Status Scale (EDSS) scores and disease duration information from the SMSC and the database for joint tasks of health insurers (SVK database) to calculate MS Severity Scores (MSSS). The SVK database collects administrative information (including EDSS) for cost approvals on behalf of specific Swiss health insurers, encompassing data from approximately 60% of all Swiss PwMS. Cost approval applications require annual re-submission by a certified neurologist. With permission of the responsible ethics committee (see below), the SMSR has been allowed to perform a probabilistic linkage between the SMSR population and the SVK database, yielding an overlap of 1021 individuals. Details on the linkage process are provided elsewhere [11].

**Fig 1:**
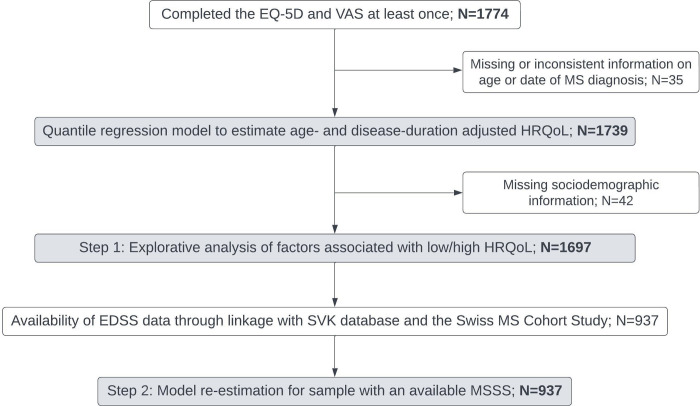
Flowchart of the study population. VAS: Visual Analogue Scale; HRQoL: Health-Related Quality of Life; EDSS: Expanded Disability Status Scale; MSSS: Multiple Sclerosis Severity Score.

This study included SMSR participants who participate in the longitudinal SMSR study, had at least one HRQoL self-assessment, and complete data on age and date of diagnosis (flowchart, Fig 1). The data were accessed for research purposes on September 25, 2023. Some authors had access to information that could identify participants. However, the data were anonymized for the analysis.

### Ethics

The SMSR and SMSC have been approved by responsible ethics committees (PB-2016-00894; BASEC-NR 2019–01027). All participants provided written informed consent. The linkage of the SVK database with the SMSR has been approved by the Ethics Committee of the Canton of Zurich (BASEC-NR 2020–00840).

### Outcome measures

The main study outcomes are HRQoL measures based on the EQ-5D survey and the visual analogue scale (VAS). The EQ-5D includes five questions on different health domains (mobility, self-care, usual activities, pain/discomfort, and anxiety/depression), convertible into a score ranging from 0 (worst HRQoL) to 100 (best HRQoL), by utilizing French value sets, which were found to be appropriate for Switzerland in an earlier study [12]. The VAS allows participants to self-rate their HRQoL on a scale from 0 (worst imaginable quality of life) to 100 (best imaginable quality of life).

### Confounders and variables of interests

In our study, we distinguished between confounders (factors that are not changeable but known to influence HRQoL) and factors of interest that are either potentially addressable in clinical care (e.g. symptoms) or potential disease course predictors (e.g. known progression risk factors, S1 Table). Pre-specified confounders handled by the quantile-based segmentation approach were age and years since diagnosis at the time of HRQoL measurement. Further analyses were also adjusted for sex and clinical MS phenotype (relapsing-remitting, primary and secondary progressive, clinically isolated syndrome). Additionally, we explored HRQoL associations with pre-specified variables of interest, including gait impairment indicator (Self-Reported Disability Status Scale (SRDSS), a proxy for the Expanded Disability Status Scale (EDSS)), self-reported current MS symptoms (dichotomous variables (yes vs. no)), six common self-reported comorbidities, specific first MS symptoms (dichotomous variables (yes vs. no), which are prognostic for a severe disease course), current DMTs, education, living situation, familial MS risk, smoking, and–where available through data linkage–MSSS, a marker for speed of MS progression, calculated according to the method of Roxburgh et al. [13]. The variables of interest were measured at the same time as HRQoL. The variables of interest and rationales for their inclusion are listed in S1 Table.

Missing information amounted to less than 3% in five categorical (clinical MS phenotype, living situation, marital status, education, smoking) and one continuous variable (Body Mass Index (BMI)), except MSSS, which was only available in 937 individuals. Categories reflecting missing or unknown information were created for the analysis of the five categorical variables. BMI was imputed using a function of age, sex, gait impairment, and self-reported diabetes or cardiovascular comorbidities. MSSS was excluded from regression model building but added to the final models in a separate analysis.

### Statistical methods

#### Classification of HRQoL groups by quartiles

For each included PwMS, the last complete HRQoL measurement was selected. First, distributional parameters, adjusted for age and years since diagnosis, were estimated. Specifically, we utilized quantile regression to estimate the 25^th^ (lowest), median, and 75^th^ (highest) quartiles using EQ-5D or VAS indices as outcomes and age and years since MS diagnosis at HRQoL assessment as explanatory variables. Subsequently, PwMS were classified as having low HRQoL (regarding EQ-5D or VAS) if an index measurement fell below the age- and disease duration adjusted lowest quartile or high HRQoL if a measured indicator was greater than the highest quartile. Index measurements between the 25^th^ and 75^th^ quartiles were considered the “normal range” within the population of PwMS. Thus, three groups per type of HRQoL index were created.

#### Exploration of factors associated with low HRQoL

We performed descriptive analyses of pre-specified sociodemographic and disease-specific factors across the three HRQoL groups. Continuous variables were summarized as medians and interquartile ranges (IQR), and categorical data as numbers and percentages. Analyses were performed separately for the VAS and EQ-5D indices. Associations were explored using multivariable multinomial regression models, with separate models for VAS and EQ-5D indicator groups. In addition to confounder control through age-and disease-duration HRQoL categorization, three additional pre-specified confounders were included in the models (sex, clinical MS phenotype, SRDSS measure for ambulation impairment). Associations with further pre-specified variables of interest were explored by adding them incrementally to the model and retaining if the Akaike Information Criterion (AIC) was reduced by two or more points. We also examined potential collinearity between the variables of the final models but did not detect any issues to address. Our group-based comparisons of health- and sociodemographic differences across HRQoL strata have the advantage of being intuitive and easily interpretable. However, the categorization comes at the cost of reduced statistical power to detect relevant differences owing to the categorization of HRQoL measures and the associated information loss. We therefore also conducted a simultaneous quantile regression for the 25^th^, 50^th^, and 75^th^ percentile as a sensitivity analysis, which uses continuous HRQoL measures [14]. Splines for age and disease duration, as well as sex, clinical MS phenotype, and a proxy measure for gait impairment (SRDSS) were included as a-prior defined confounders, and other variables were added incrementally. Model variance was estimated via bootstrapping with 100 repetitions, and variables were selected on the basis of p-values <0.05.

#### Analysis of discordant VAS and EQ-5D

We examined individuals with discordant VAS and EQ-5D assessments, namely VAS ratings markedly lower or higher than EQ-5D indices. Discordance was defined as separation by at least two quantiles. Individuals with a high EQ-5D-low VAS were identified if they had an EQ-5D in the highest quartile and a VAS in the lowest two quartiles or if they had an EQ-5D in the highest two quartiles and a VAS in the lowest quartile. Individuals with a low EQ-5D-high VAS were identified if they had an EQ-5D in the lowest quartile and a VAS in the highest two quartiles or if they had an EQ-5D in the lowest two quartiles and a VAS in the highest quartile. Descriptive and multivariable regression analyses were performed as described above.

Analyses were conducted using Stata SE Version 16.1 (Stata Corp., College Station, TX, USA).

## Results

### Description of study population

Our study included 1697 PwMS, of whom 73.1% were female (Table 1, column 2). The median age was 49 (IQR 38–58) years, and the median time since diagnosis was 9 (IQR 4–17) years. 68.4% of PwMS reported having relapsing-remitting MS, 4.1% a clinically isolated syndrome, a total of 26.7% a primary or secondary progressive MS, and for 0.8%, clinical MS phenotype was unclear. Median EQ-5D and VAS were 90.7 and 80 points.

**Table 1:** Description of the study population (column 2) and HRQoL quantile groups for EQ-5D (columns 3–5) and VAS (columns 6–8).

		EQ-5D index			Visual analogue scale		
	Full sample	lowest quartile	middle quartiles	highest quartile	lowest quartile	middle quartiles	highest quartile
	N=1697	N=417	N=858	N=422	N=415	N=861	N=421
Sociodemographic characteristics							
Female sex	1241 (73.1)	287 (68.8)	645 (75.2)	309 (73.2)	295 (71.1)	634 (73.6)	312 (74.1)
Male sex	456 (26.9)	130 (31.2)	213 (24.8)	113 (26.8)	120 (28.9)	227 (26.4)	109 (25.9)
Age, median [IQR]	49 [38; 58]	49 [39; 58]	49 [38; 58]	49 [38; 57]	49 [38; 58]	49 [39; 58]	49 [38; 57]
Swiss citizen	1519 (89.5)	371 (89)	768 (89.5)	380 (90)	361 (87)	769 (89.3)	389 (92.4)
Currently employed	1034 (60.9)	181 (43.4)	543 (63.3)	310 (73.5)	194 (46.7)	540 (62.7)	300 (71.3)
Disability benefits							
*Does not receive disability insurance*	1020 (60.1)	133 (31.9)	517 (60.3)	370 (87.7)	129 (31.1)	537 (62.4)	354 (84.1)
*Has applied for disability insurance*	106 (6.2)	51 (12.2)	52 (6.1)	3 (0.7)	64 (15.4)	38 (4.4)	4 (1)
*Does receive disability insurance*	571 (33.6)	233 (55.9)	289 (33.7)	49 (11.6)	222 (53.5)	286 (33.2)	63 (15)
Education							
*Mandatory schooling completed*	65 (3.8)	21 (5)	31 (3.6)	13 (3.1)	23 (5.5)	29 (3.4)	13 (3.1)
*Apprenticeship*	688 (40.5)	201 (48.2)	346 (40.3)	141 (33.4)	184 (44.3)	346 (40.2)	158 (37.5)
*Highschool*	156 (9.2)	32 (7.7)	82 (9.6)	42 (10)	32 (7.7)	90 (10.5)	34 (8.1)
*Higher professional education*	248 (14.6)	61 (14.6)	125 (14.6)	62 (14.7)	60 (14.5)	121 (14.1)	67 (15.9)
*University degree*	466 (27.5)	83 (19.9)	234 (27.3)	149 (35.3)	93 (22.4)	239 (27.8)	134 (31.8)
*Other or unknown education status*	74 (4.4)	19 (4.6)	40 (4.7)	15 (3.6)	23 (5.5)	36 (4.2)	15 (3.6)
Living situation							
*Lives at clinic/nursing home*	6 (0.4)	5 (1.2)	1 (0.1)	0 (0)	3 (0.7)	3 (0.3)	0 (0)
*Lives with family*	502 (29.6)	121 (29)	256 (29.8)	125 (29.6)	117 (28.2)	255 (29.6)	130 (30.9)
*Lives with friends/relatives*	44 (2.6)	13 (3.1)	17 (2)	14 (3.3)	9 (2.2)	21 (2.4)	14 (3.3)
*Lives alone*	372 (21.9)	89 (21.3)	193 (22.5)	90 (21.3)	94 (22.7)	192 (22.3)	86 (20.4)
*Lives with parents*	36 (2.1)	8 (1.9)	18 (2.1)	10 (2.4)	8 (1.9)	18 (2.1)	10 (2.4)
*Lives with spouse/partner*	698 (41.1)	173 (41.5)	348 (40.6)	177 (41.9)	175 (42.2)	349 (40.5)	174 (41.3)
*Other or unknown living situation*	39 (2.3)	8 (1.9)	25 (2.9)	6 (1.4)	9 (2.2)	23 (2.7)	7 (1.7)
Marital status							
*Divorced*	185 (10.9)	56 (13.4)	89 (10.4)	40 (9.5)	63 (15.2)	81 (9.4)	41 (9.7)
*Married*	852 (50.2)	208 (49.9)	437 (50.9)	207 (49.1)	202 (48.7)	437 (50.8)	213 (50.6)
*In registered partnership*	36 (2.1)	11 (2.6)	20 (2.3)	5 (1.2)	11 (2.7)	21 (2.4)	4 (1)
*Separated*	41 (2.4)	15 (3.6)	18 (2.1)	8 (1.9)	14 (3.4)	18 (2.1)	9 (2.1)
*Unmarried*	513 (30.2)	110 (26.4)	254 (29.6)	149 (35.3)	108 (26)	268 (31.1)	137 (32.5)
*Widowed*	45 (2.7)	12 (2.9)	25 (2.9)	8 (1.9)	9 (2.2)	23 (2.7)	13 (3.1)
*Unknown marital status*	25 (1.5)	5 (1.2)	15 (1.7)	5 (1.2)	8 (1.9)	13 (1.5)	4 (1)
MS disease characteristics							
Years since diagnosis, median [IQR]	9 [4; 17]	9 [4; 17]	9 [4; 17]	9 [4; 17]	9 [4; 17]	9 [4; 17]	9 [4; 17]
Age at diagnosis, median [IQR]	37 [28; 45]	37 [28; 45]	36 [29; 45]	37 [28; 45]	36 [28; 45]	37 [28; 45]	37 [29; 45]
Clinical MS phenotype							
*Clinically Isolated Syndrome (CIS)*	70 (4.1)	9 (2.2)	28 (3.3)	33 (7.8)	10 (2.4)	36 (4.2)	24 (5.7)
*Primary progressive MS (PPMS)*	180 (10.6)	68 (16.3)	97 (11.3)	15 (3.6)	65 (15.7)	93 (10.8)	22 (5.2)
*Relapsing-remitting MS (RRMS)*	1161 (68.4)	221 (53)	596 (69.5)	344 (81.5)	241 (58.1)	590 (68.5)	330 (78.4)
*Secondary progressive MS (SPMS)*	273 (16.1)	117 (28.1)	128 (14.9)	28 (6.6)	96 (23.1)	135 (15.7)	42 (10)
*Unclear clinical MS phenotype*	13 (0.8)	2 (0.5)	9 (1.0)	2 (0.5)	3 (0.7)	7 (0.8)	3 (0.7)
MS Severity Score, N, median [IQR]	937; 5.2 [2.1; 7.4]	229; 7.2 [4.5; 9]	469; 5.3 [3.1; 7.3]	239; 2.8 [1.3; 5.1]	235; 6.6 [4.1; 8.6]	460; 5.1 [2.8; 7.2]	242; 3.4 [1.7; 6.2]
Adjusted MS Severity Score, N, median [IQR]	937; 5.1 [2.3; 7.6]	229; 7.5 [4.9; 8.9]	469; 5.3 [3; 7.3]	239; 2.5 [1.1; 4.9]	235; 7 [4.2; 8.8]	460; 5.1 [2.6; 7.2]	242; 3.3 [1.3; 5.9]
Health-Related Quality of Life measurements							
EQ-5D index, median [IQR]	90.7 [80; 96.2]	61.5 [43.2; 78.2]	90.7 [86.2; 94.5]	100 [96.7; 100]	71.2 [54.2; 87]	90.5 [84.6; 95.8]	97.8 [94; 100]
Visual Analogue Scale, median [IQR]	80 [60; 90]	50 [39; 70]	80 [65; 90]	90 [85; 98]	40 [32; 50]	80 [70; 85]	95 [90; 99]
Current symptom burden							
Balance problems	606 (35.7)	224 (53.7)	332 (38.7)	50 (11.8)	227 (54.7)	308 (35.8)	71 (16.9)
Bladder problems	494 (29.1)	190 (45.6)	267 (31.1)	37 (8.8)	189 (45.5)	242 (28.1)	63 (15)
Depression	216 (12.7)	116 (27.8)	96 (11.2)	4 (0.9)	111 (26.7)	96 (11.1)	9 (2.1)
Fatigue	921 (54.3)	320 (76.7)	514 (59.9)	87 (20.6)	330 (79.5)	464 (53.9)	127 (30.2)
Gait problems	594 (35)	222 (53.2)	334 (38.9)	38 (9)	229 (55.2)	304 (35.3)	61 (14.5)
Gastrointestinal problems	388 (22.9)	182 (43.6)	185 (21.6)	21 (5)	161 (38.8)	198 (23)	29 (6.9)
Memory problems	371 (21.9)	165 (39.6)	179 (20.9)	27 (6.4)	176 (42.4)	159 (18.5)	36 (8.6)
Pain	504 (29.7)	223 (53.5)	256 (29.8)	25 (5.9)	216 (52)	238 (27.6)	50 (11.9)
Paresthesia	749 (44.1)	250 (60)	401 (46.7)	98 (23.2)	254 (61.2)	384 (44.6)	111 (26.4)
Spasms	495 (29.2)	210 (50.4)	263 (30.7)	22 (5.2)	201 (48.4)	246 (28.6)	48 (11.4)
Tremors	178 (10.5)	94 (22.5)	77 (9)	7 (1.7)	80 (19.3)	77 (8.9)	21 (5)
Muscle weakness	550 (32.4)	245 (58.8)	275 (32.1)	30 (7.1)	239 (57.6)	264 (30.7)	47 (11.2)
Number of MS Symptoms: median [IQR]	4 [0; 8]	8 [5; 11]	4 [1; 7]	0 [0; 2]	8 [5; 11]	4 [1; 7]	1 [0; 3]
*0–2 Symptoms*	676 (39.8)	54 (12.9)	290 (33.8)	332 (78.7)	59 (14.2)	327 (38)	290 (68.9)
*3–6 Symptoms*	470 (27.7)	103 (24.7)	289 (33.7)	78 (18.5)	102 (24.6)	276 (32.1)	92 (21.9)
*7 or more Symptoms*	551 (32.5)	260 (62.4)	279 (32.5)	12 (2.8)	254 (61.2)	258 (30)	39 (9.3)
Ambulatory impairments							
Self-reported disability status scale (SRDSS)							
*SRDSS 0–3*.*5*	1193 (70.3)	176 (42.2)	621 (72.4)	396 (93.8)	200 (48.2)	619 (71.9)	374 (88.8)
*SRDSS 4–6*.*5*	349 (20.6)	126 (30.2)	200 (23.3)	23 (5.5)	145 (34.9)	173 (20.1)	31 (7.4)
*SRDSS 7 and higher*	155 (9.1)	115 (27.6)	37 (4.3)	3 (0.7)	70 (16.9)	69 (8)	16 (3.8)
Uses a wheelchair	257 (15.1)	161 (38.6)	90 (10.5)	6 (1.4)	120 (28.9)	117 (13.6)	20 (4.8)
Uses a cane or crutches	271 (16)	103 (24.7)	150 (17.5)	18 (4.3)	116 (28)	132 (15.3)	23 (5.5)
Uses a rollator	146 (8.6)	68 (16.3)	75 (8.7)	3 (0.7)	77 (18.6)	57 (6.6)	12 (2.9)
Current disease-modifying treatments							
*Oral disease-modifying treatment*	571 (33.6)	118 (28.3)	291 (33.9)	162 (38.4)	135 (32.5)	278 (32.3)	158 (37.5)
*Monoclonal disease-modifying treatment*	334 (19.7)	107 (25.7)	177 (20.6)	50 (11.8)	97 (23.4)	179 (20.8)	58 (13.8)
*Injectable disease-modifying treatment*	235 (13.8)	32 (7.7)	120 (14)	83 (19.7)	41 (9.9)	120 (13.9)	74 (17.6)
*Other disease-modifying treatment*	30 (1.8)	14 (3.4)	11 (1.3)	5 (1.2)	11 (2.7)	15 (1.7)	4 (1)
*No disease-modifying treatment*	527 (31.1)	146 (35)	259 (30.2)	122 (28.9)	131 (31.6)	269 (31.2)	127 (30.2)
Risk factors for MS onset and progression							
Reported MS cases in bloodline relatives	239 (14.1)	60 (14.4)	127 (14.8)	52 (12.3)	61 (14.7)	118 (13.7)	60 (14.3)
Visual disturbances as first symptom	680 (40.1)	175 (42)	339 (39.5)	166 (39.3)	179 (43.1)	336 (39)	165 (39.2)
Paresthesia as first symptom	1000 (58.9)	239 (57.3)	495 (57.7)	266 (63)	239 (57.6)	495 (57.5)	266 (63.2)
Gait problems as first symptom	526 (31)	169 (40.5)	269 (31.4)	88 (20.9)	150 (36.1)	281 (32.6)	95 (22.6)
Spasms as first symptom	142 (8.4)	49 (11.8)	70 (8.2)	23 (5.5)	47 (11.3)	75 (8.7)	20 (4.8)
Body Mass Index, N, median [IQR]	1641; 24.1 [21.4; 27.3]	399; 24.9 [21.6;28.9]	834; 23.9 [21.3; 27.3]	408; 23.4 [21.4; 25.9]	398; 24.4 [21.3; 28.4]	835; 24.2 [21.5; 27.4]	408; 23.7 [21.4; 26.2]
Smoking status							
*Unknown*	11 (0.6)	4 (1)	6 (0.7)	1 (0.2)	4 (1)	5 (0.6)	2 (0.5)
*Never smoked*	738 (43.5)	165 (39.6)	360 (42)	213 (50.5)	151 (36.4)	381 (44.3)	206 (48.9)
*Past smoker*	364 (21.4)	112 (26.9)	187 (21.8)	65 (15.4)	114 (27.5)	175 (20.3)	75 (17.8)
*Current smoker*	584 (34.4)	136 (32.6)	305 (35.5)	143 (33.9)	146 (35.2)	300 (34.8)	138 (32.8)
Self-reported comorbidities							
Mononucleosis	246 (14.5)	54 (12.9)	137 (16)	55 (13)	58 (14)	124 (14.4)	64 (15.2)
High blood pressure	219 (12.9)	65 (15.6)	109 (12.7)	45 (10.7)	57 (13.7)	113 (13.1)	49 (11.6)
Cancer	44 (2.6)	9 (2.2)	27 (3.1)	8 (1.9)	13 (3.1)	21 (2.4)	10 (2.4)
Diabetes Type 1	7 (0.4)	3 (0.7)	4 (0.5)	0 (0)	3 (0.7)	3 (0.3)	1 (0.2)
Diabetes Type 2	28 (1.6)	15 (3.6)	9 (1)	4 (0.9)	10 (2.4)	11 (1.3)	7 (1.7)
Cardiovascular problems	68 (4)	20 (4.8)	34 (4)	14 (3.3)	18 (4.3)	35 (4.1)	15 (3.6)

Values are presented as numbers and percentages unless otherwise specified.

### Factors associated with low EQ-5D

The quantile-based EQ-5D classification yielded 417 PwMS in the lowest quartile, 858 in the middle quartiles, and 422 in the highest quartile (Table 1, columns 3–5). Median EQ-5D across the groups were 61.5 (lowest quartile), 90.7 (middle quartiles), and 100 points (highest quartile). By design, the three groups were similar regarding age and time since diagnosis. The lowest quartile group included higher percentages of male PwMS (31.2% vs. 26.8%) and persons with primary and secondary progressive MS (44.4% vs. 10.2%) compared to the highest quartile group. The lowest quartile group had the highest number of symptoms (median of 8 vs. 0) and the highest percentage of individuals with severe gait impairment (27.6% with SRDSS 7 or greater vs. 0.7) compared to the highest quartile group. However, descriptive analyses revealed no marked differences in sociodemographic (e.g., education, marital status) or MS risk factors (e.g., smoking, familial MS risk).

As shown in Table 2, testing the pre-specified characteristics in a multivariable multinomial regression model identified disability benefit status (Relative Risk Ratio, RRR [95% Confidence Interval, 95%CI] compared with middle quartiles; 1.52 [1.11–2.08]), severe gait impairment measured by SRDSS of 7 or more (12.69 [7.29–22.11]), BMI (1.05 [1.03–1.08] per score increase), as well as different symptoms (depression (2.37 [1.64–3.42]), memory problems (1.46 [1.04–2.05]), pain (1.69 [1.22–2.35]), and muscle weakness (1.50 [1.07–2.08])) as independently associated factors with low EQ-5D. Based on the AIC, additional factors with less frequent accumulation in the highest quartile group, namely a number of 7 or more MS symptoms (0.16 [0.07–0.36]), past smoking (0.60 [0.41–0.87]), or the presence of fatigue (0.48 [0.33–0.70]), were retained in the model.

**Table 2:** Multivariable multinomial regression to identify associations with lower-than-expected and higher-than-expected EQ-5D.

	Univariable, N=1697		Multivariable, N=1697		Multivariable incl. MSSS, N=937	
	lowest quartile	highest quartile	lowest quartile	highest quartile	lowest quartile	highest quartile
	RRR [95%CI]	RRR [95%CI]	RRR [95%CI]	RRR [95%CI]	RRR [95%CI]	RRR [95%CI]
	N=417	N=422	N=417	N=422	N=229	N=239
Sociodemographic characteristics						
Female sex	Reference	Reference	Reference	Reference	Reference	Reference
Male sex	1.37 [1.06; 1.78]	1.11 [0.85; 1.44]	1.15 [0.84; 1.58]	1.42 [1.02; 1.96]	1.07 [0.68; 1.70]	1.13 [0.72; 1.77]
Swiss citizen	0.95 [0.65; 1.38]	1.06 [0.72; 1.56]				
Currently employed	0.44 [0.35; 0.56]	1.61 [1.24; 2.08]	0.90 [0.66; 1.22]	0.58 [0.41; 0.82]	0.85 [0.55; 1.30]	0.56 [0.33; 0.93]
Disability benefits						
*Does not receive disability insurance*	Reference	Reference	Reference	Reference	Reference	Reference
*Has applied for disability insurance*	3.81 [2.48; 5.86]	0.08 [0.02; 0.26]	2.50 [1.54; 4.08]	0.19 [0.06; 0.64]	4.10 [2.03; 8.25]	0.15 [0.02; 1.16]
*Does receive disability insurance*	3.13 [2.42; 4.05]	0.24 [0.17; 0.33]	1.52 [1.11; 2.08]	0.48 [0.32; 0.71]	2.22 [1.41; 3.48]	0.37 [0.21; 0.66]
Education						
*Mandatory schooling completed*	Reference	Reference				
*Apprenticeship*	0.86 [0.48; 1.53]	0.97 [0.49; 1.91]				
*Highschool*	0.58 [0.29; 1.15]	1.22 [0.58; 2.58]				
*Higher professional education*	0.72 [0.38; 1.36]	1.18 [0.58; 2.42]				
*University degree*	0.52 [0.29; 0.96]	1.52 [0.77; 3.00]				
*Other or unknown education status*	0.70 [0.32; 1.53]	0.89 [0.37; 2.15]				
Living situation						
*Lives with family*	Reference	Reference				
*Lives with friends/relatives*	1.62 [0.76; 3.44]	1.69 [0.81; 3.53]				
*Lives alone*	0.98 [0.70; 1.36]	0.96 [0.69; 1.33]				
*Lives with parents*	1.06 [0.53; 2.13]	0.47 [0.19; 1.18]				
*Lives with spouse/partner*	0.94 [0.40; 2.22]	1.14 [0.51; 2.54]				
*Other or unknown living situation*	1.05 [0.79; 1.40]	1.04 [0.79; 1.38]				
Marital status						
*Divorced*	Reference	Reference				
*Married*	0.76 [0.52; 1.10]	1.05 [0.70; 1.59]				
*In registered partnership*	0.53 [0.18; 1.54]	0.74 [0.25; 2.18]				
*Separated*	0.87 [0.39; 1.96]	0.56 [0.19; 1.59]				
*Unmarried*	1.32 [0.62; 2.84]	0.99 [0.40; 2.46]				
*Widowed*	0.69 [0.46; 1.03]	1.31 [0.85; 2.00]				
*Unknown marital status*	0.76 [0.35; 1.64]	0.71 [0.30; 1.72]				
MS disease characteristics						
Clinical MS phenotype						
*Clinically Isolated Syndrome (CIS)*	0.87 [0.40; 1.87]	2.04 [1.21; 3.44]	1.28 [0.52; 3.16]	1.43 [0.79; 2.57]	4.48 [1.18; 16.93]	1.42 [0.56; 3.59]
*Primary progressive MS (PPMS)*	1.89 [1.34; 2.67]	0.27 [0.15; 0.47]	1.02 [0.64; 1.62]	0.52 [0.26; 1.02]	0.62 [0.29; 1.30]	0.75 [0.23; 2.34]
*Relapsing-remitting MS (RRMS)*	Reference	Reference	Reference	Reference	Reference	Reference
*Secondary progressive MS (SPMS)*	2.47 [1.84; 3.31]	0.38 [0.25; 0.58]	1.11 [0.72; 1.73]	0.81 [0.45; 1.44]	0.94 [0.50; 1.76]	0.87 [0.36; 2.07]
*Unspecified phase*	0.60 [0.13; 2.80]	0.39 [0.08; 1.79]	0.30 [0.05; 1.97]	0.67 [0.11; 4.17]	0.34 [0.04; 3.21]	0.00 [0.00; .]
MS Severity Score (MSSS)	1.23 [1.16; 1.31]	0.78 [0.73; 0.83]	n.d.	n.d.	1.14 [1.05; 1.23]	0.88 [0.81; 0.95]
Current symptom burden						
Balance problems	1.84 [1.45; 2.33]	0.21 [0.15; 0.29]				
Bladder problems	1.85 [1.46; 2.36]	0.21 [0.15; 0.31]				
Depression	3.06 [2.26; 4.14]	0.08 [0.03; 0.21]	2.37 [1.64; 3.42]	0.27 [0.09; 0.80]	2.58 [1.50; 4.45]	0.27 [0.06; 1.27]
Fatigue	2.21 [1.69; 2.88]	0.17 [0.13; 0.23]	1.28 [0.85; 1.91]	0.48 [0.33; 0.70]	1.33 [0.74; 2.37]	0.44 [0.26; 0.73]
Gait problems	1.79 [1.41; 2.26]	0.16 [0.11; 0.22]				
Gastrointestinal problems	2.82 [2.19; 3.63]	0.19 [0.12; 0.30]				
Memory problems	2.48 [1.92; 3.21]	0.26 [0.17; 0.40]	1.46 [1.04; 2.05]	1.28 [0.74; 2.20]	1.37 [0.85; 2.23]	0.96 [0.47; 1.96]
Pain	2.70 [2.12; 3.44]	0.15 [0.10; 0.23]	1.69 [1.22; 2.35]	0.58 [0.35; 0.98]	1.22 [0.77; 1.94]	0.71 [0.35; 1.46]
Paresthesia	1.71 [1.35; 2.16]	0.34 [0.27; 0.45]				
Spasms	2.30 [1.80; 2.92]	0.12 [0.08; 0.20]				
Tremors	2.95 [2.13; 4.10]	0.17 [0.08; 0.37]				
Muscle weakness	3.02 [2.37; 3.85]	0.16 [0.11; 0.24]	1.50 [1.07; 2.08]	0.79 [0.48; 1.32]	1.65 [1.02; 2.68]	0.79 [0.38; 1.65]
Number of MS Symptoms						
*0–2 Symptoms*	Reference	Reference	Reference	Reference	Reference	Reference
*3–6 Symptoms*	1.91 [1.33; 2.76]	0.24 [0.18; 0.32]	0.99 [0.61; 1.61]	0.52 [0.34; 0.78]	1.37 [0.69; 2.70]	0.71 [0.40; 1.26]
*7 or more Symptoms*	5.00 [3.58; 7.00]	0.04 [0.02; 0.07]	1.11 [0.61; 2.01]	0.16 [0.07; 0.36]	1.73 [0.73; 4.09]	0.28 [0.09; 0.89]
Ambulatory impairments						
Self-reported disability status scale (SRDSS)						
*SRDSS 0–3*.*5*	Reference	Reference	Reference	Reference	Reference	Reference
*SRDSS 4–6*.*5*	2.22 [1.68; 2.94]	0.18 [0.12; 0.28]	1.67 [1.15; 2.43]	0.31 [0.18; 0.56]	1.12 [0.66; 1.92]	0.17 [0.07; 0.45]
*SRDSS 7 and higher*	10.97 [7.30; 16.47]	0.13 [0.04; 0.42]	12.69 [7.29; 22.11]	0.17 [0.05; 0.60]	13.35 [5.76; 30.91]	0.29 [0.05; 1.55]
Uses a wheelchair	5.37 [4.00; 7.20]	0.12 [0.05; 0.28]				
Uses a cane or crutches	1.55 [1.17; 2.06]	0.21 [0.13; 0.35]				
Uses a rollator	2.03 [1.43; 2.89]	0.07 [0.02; 0.24]				
Current disease-modifying treatments						
*Oral disease-modifying treatment*	Reference	Reference	Reference	Reference	Reference	Reference
*Monoclonal disease-modifying treatment*	1.49 [1.08; 2.06]	0.51 [0.35; 0.73]	1.24 [0.85; 1.80]	0.65 [0.43; 0.99]	1.87 [1.11; 3.15]	0.58 [0.33; 1.00]
*Injectable disease-modifying treatment*	0.66 [0.42; 1.03]	1.24 [0.88; 1.74]	0.51 [0.30; 0.85]	1.18 [0.79; 1.76]	0.62 [0.30; 1.25]	1.20 [0.70; 2.05]
*Other disease-modifying treatment*	3.14 [1.38; 7.11]	0.82 [0.28; 2.39]	1.41 [0.52; 3.83]	1.48 [0.41; 5.36]	1.42 [0.37; 5.53]	2.41 [0.54; 10.77]
*No disease-modifying treatment*	1.39 [1.04; 1.87]	0.85 [0.63; 1.13]	0.71 [0.48; 1.04]	1.27 [0.89; 1.83]	0.86 [0.49; 1.50]	1.23 [0.74; 2.05]
Risk factors for MS onset and progression						
Reported MS cases in bloodline relatives	0.97 [0.69; 1.35]	0.81 [0.57; 1.14]				
Visual disturbances as first symptom	1.11 [0.87; 1.40]	0.99 [0.78; 1.26]				
Paresthesia as first symptom	0.98 [0.78; 1.25]	1.25 [0.98; 1.59]				
Gait problems as first symptom	1.49 [1.17; 1.90]	0.58 [0.44; 0.76]				
Spasms as first symptom	1.50 [1.02; 2.20]	0.65 [0.40; 1.06]				
Body Mass Index	1.04 [1.02; 1.07]	0.97 [0.94; 0.99]	1.05 [1.03; 1.08]	0.95 [0.93; 0.98]	1.03 [1.00; 1.07]	0.96 [0.92; 1.00]
Smoking status						
*Unknown*	1.45 [0.41; 5.22]	0.28 [0.03; 2.36]	1.14 [0.25; 5.23]	0.29 [0.03; 2.84]	1.15 [0.05; 26.41]	0.32 [0.03; 3.77]
*Never smoked*	Reference	Reference	Reference	Reference	Reference	Reference
*Past smoker*	1.31 [0.97; 1.76]	0.59 [0.42; 0.82]	1.30 [0.92; 1.85]	0.60 [0.41; 0.87]	1.30 [0.79; 2.14]	0.71 [0.43; 1.18]
*Current smoker*	0.97 [0.74; 1.28]	0.79 [0.61; 1.03]	0.85 [0.62; 1.18]	0.83 [0.60; 1.13]	0.88 [0.55; 1.40]	0.82 [0.53; 1.26]
Self-reported comorbidities						
Mononucleosis	0.78 [0.56; 1.10]	0.79 [0.56; 1.10]				
High blood pressure	0.68 [0.32; 1.46]	0.59 [0.27; 1.32]				
Cancer	1.27 [0.91; 1.77]	0.82 [0.57; 1.19]				
Diabetes Type 1	1.55 [0.34; 6.94]	0.00 [0.00; Inf]				
Diabetes Type 2	3.52 [1.53; 8.11]	0.90 [0.28; 2.95]				
Cardiovascular problems	1.22 [0.69; 2.15]	0.83 [0.44; 1.57]				

Re-calculating the final model only including individuals with a MSSS (n = 937), the factors associated with low EQ-5D remained similar. Also, the MSSS exhibited a marked association with low EQ-5D (1.14 [1.05–1.23]).

The simultaneous quantile regression identified fewer statistically significant factors to exert a differential impact on three quantiles (S2 Table). Compared with the main analysis, SRDSS strata, symptom load, depression and memory problems were also flagged as factors with a more pronounced effect in PwMS with low HRQoL. In addition, gait impairments or spams as first symptoms were newly identified as factors associated with low HRQoL.

### Factors associated with low VAS

The VAS-based classification yielded groups of 415 (lowest quartile; median VAS 40 points), 861 (middle quartiles; 80 points), and 421 (highest quartile; 95 points) persons (Table 1, columns 6–8). Like the EQ-5D analysis, the lowest VAS quartile group had the highest proportion of persons with an SRDSS of 7 or greater (16.9%), the largest median number of symptoms (8), or persons with primary or secondary progressive MS (38.8%) compared with other quartile groups.

Multivariable regression (Table 3) identified receiving disability benefits (RRR 1.86 [95% CI 1.38–2.50]), SRDSS of 7 or more (3.14 [1.91–5.16]), and the following MS symptoms: depression (1.61 [1.12–2.31]), fatigue (1.80 [1.23–2.63]), memory problems (1.77 [1.28–2.46]), pain (1.40 [1.02–1.91]), and muscle weakness (1.40 [1.02–1.92]) to be associated with the lowest VAS quartile (compared with middle quartiles). Moreover, Swiss citizenship was associated with the highest VAS quartile (1.76 [1.13–2.75]).

**Table 3:** Multivariable multinomial regression to identify associations with lower-than-expected and higher-than-expected VAS.

	Univariable, N=1697		Multivariable, N=1697		Multivariable incl. MSSS, N=937	
	lowest quartile	highest quartile	lowest quartile	highest quartile	lowest quartile	highest quartile
	RRR [95%CI]	RRR [95%CI]	RRR [95%CI]	RRR [95%CI]	RRR [95%CI]	RRR [95%CI]
	N=417	N=422	N=417	N=422	N=235	N=242
Sociodemographic characteristics						
Female sex	Reference	Reference	Reference	Reference	Reference	Reference
Male sex	1.14 [0.88; 1.47]	0.98 [0.75; 1.27]	0.99 [0.73; 1.33]	1.07 [0.80; 1.44]	0.96 [0.62; 1.47]	1.16 [0.78; 1.74]
Swiss citizen	0.80 [0.56; 1.14]	1.45 [0.96; 2.21]	0.64 [0.43; 0.97]	1.76 [1.13; 2.75]	0.63 [0.34; 1.18]	1.30 [0.70; 2.41]
Currently employed	0.52 [0.41; 0.66]	1.47 [1.15; 1.90]				
Disability benefits						
*Does not receive disability insurance*						
*Has applied for disability insurance*	7.01 [4.49; 10.94]	0.16 [0.06; 0.45]	4.33 [2.68; 7.02]	0.26 [0.09; 0.75]	6.37 [3.15; 12.88]	0.14 [0.02; 1.12]
*Does receive disability insurance*	3.23 [2.49; 4.19]	0.33 [0.25; 0.45]	1.86 [1.38; 2.50]	0.53 [0.38; 0.74]	2.31 [1.51; 3.51]	0.47 [0.29; 0.74]
Education						
*Mandatory schooling completed*	Reference	Reference				
*Apprenticeship*	0.67 [0.38; 1.19]	1.02 [0.52; 2.01]				
*Highschool*	0.45 [0.23; 0.88]	0.84 [0.39; 1.81]				
*Higher professional education*	0.63 [0.33; 1.17]	1.24 [0.60; 2.54]				
*University degree*	0.49 [0.27; 0.89]	1.25 [0.63; 2.49]				
*Other or unknown education status*	0.81 [0.38; 1.72]	0.93 [0.38; 2.26]				
Living situation						
*Lives with family*	Reference	Reference				
*Lives with friends/relatives*	0.93 [0.42; 2.10]	1.31 [0.64; 2.66]				
*Lives alone*	1.07 [0.77; 1.48]	0.88 [0.63; 1.22]				
*Lives with parents*	1.01 [0.49; 2.06]	0.53 [0.22; 1.25]				
*Lives with spouse/partner*	0.97 [0.41; 2.29]	1.09 [0.49; 2.43]				
*Other or unknown living situation*	1.09 [0.82; 1.45]	0.98 [0.74; 1.29]				
Marital status						
*Divorced*	Reference	Reference				
*Married*	0.59 [0.41; 0.86]	0.96 [0.64; 1.45]				
*In registered partnership*	0.79 [0.31; 2.03]	0.61 [0.19; 1.98]				
*Separated*	0.67 [0.30; 1.50]	0.38 [0.12; 1.17]				
*Unmarried*	1.00 [0.46; 2.16]	0.99 [0.41; 2.39]				
*Widowed*	0.52 [0.35; 0.77]	1.01 [0.66; 1.55]				
*Unknown marital status*	0.50 [0.22; 1.16]	1.12 [0.51; 2.43]				
MS disease characteristics						
Clinical MS phenotype						
*Clinically Isolated Syndrome (CIS)*	0.68 [0.33; 1.39]	1.19 [0.70; 2.03]	1.07 [0.47; 2.41]	0.88 [0.50; 1.54]	2.28 [0.67; 7.79]	1.21 [0.51; 2.87]
*Primary progressive MS (PPMS)*	1.71 [1.21; 2.43]	0.42 [0.26; 0.69]	1.11 [0.72; 1.71]	0.78 [0.45; 1.35]	0.56 [0.28; 1.14]	0.57 [0.23; 1.45]
*Relapsing-remitting MS (RRMS)*	Reference	Reference	Reference	Reference	Reference	Reference
*Secondary progressive MS (SPMS)*	1.74 [1.29; 2.35]	0.56 [0.38; 0.81]	0.95 [0.63; 1.42]	1.20 [0.74; 1.94]	0.84 [0.47; 1.49]	1.00 [0.49; 2.04]
*Unspecified phase*	1.05 [0.27; 4.09]	0.77 [0.20; 2.98]	0.73 [0.17; 3.12]	1.54 [0.36; 6.63]	1.43 [0.24; 8.55]	0.99 [0.09; 11.11]
MS Severity Score (MSSS)	1.17 [1.11; 1.24]	0.88 [0.83; 0.93]	n.d.	n.d.	1.07 [0.99; 1.15]	0.98 [0.92; 1.05]
Current symptom burden						
Balance problems	2.17 [1.71; 2.75]	0.36 [0.27; 0.49]				
Bladder problems	2.14 [1.68; 2.73]	0.45 [0.33; 0.61]				
Depression	2.91 [2.15; 3.94]	0.17 [0.09; 0.35]	1.61 [1.12; 2.31]	0.38 [0.18; 0.79]	1.38 [0.81; 2.36]	0.43 [0.14; 1.30]
Fatigue	3.32 [2.53; 4.37]	0.37 [0.29; 0.47]	1.80 [1.23; 2.63]	0.84 [0.60; 1.19]	1.85 [1.08; 3.16]	0.93 [0.58; 1.49]
Gait problems	2.26 [1.78; 2.86]	0.31 [0.23; 0.42]				
Gastrointestinal problems	2.12 [1.65; 2.73]	0.25 [0.16; 0.37]				
Memory problems	3.25 [2.51; 4.22]	0.41 [0.28; 0.61]	1.77 [1.28; 2.46]	1.00 [0.63; 1.57]	1.76 [1.11; 2.79]	1.11 [0.60; 2.04]
Pain	2.84 [2.23; 3.63]	0.35 [0.25; 0.49]	1.40 [1.02; 1.91]	0.86 [0.57; 1.30]	0.97 [0.62; 1.52]	1.06 [0.58; 1.91]
Paresthesia	1.96 [1.54; 2.49]	0.44 [0.34; 0.57]	1.28 [0.95; 1.72]	0.72 [0.53; 0.97]	1.29 [0.85; 1.97]	0.61 [0.40; 0.94]
Spasms	2.35 [1.84; 2.99]	0.32 [0.23; 0.45]				
Tremors	2.43 [1.73; 3.41]	0.53 [0.33; 0.88]				
Muscle weakness	3.07 [2.41; 3.91]	0.28 [0.20; 0.40]	1.40 [1.02; 1.92]	0.73 [0.48; 1.10]	1.66 [1.05; 2.62]	0.57 [0.30; 1.08]
Number of MS Symptoms						
*0–2 Symptoms*	Reference	Reference	Reference	Reference	Reference	Reference
*3–6 Symptoms*	2.05 [1.43; 2.93]	0.38 [0.28; 0.50]	0.73 [0.45; 1.17]	0.66 [0.44; 0.98]	0.59 [0.31; 1.13]	0.60 [0.35; 1.03]
*7 or more Symptoms*	5.46 [3.93; 7.57]	0.17 [0.12; 0.25]	0.77 [0.42; 1.40]	0.53 [0.28; 1.00]	0.99 [0.44; 2.26]	0.42 [0.17; 1.06]
Ambulatory impairments						
Self-reported disability status scale (SRDSS)						
*SRDSS 0–3*.*5*	Reference	Reference	Reference	Reference	Reference	Reference
*SRDSS 4–6*.*5*	2.59 [1.98; 3.41]	0.30 [0.20; 0.44]	2.02 [1.41; 2.89]	0.46 [0.28; 0.76]	1.71 [1.03; 2.86]	0.56 [0.28; 1.13]
*SRDSS 7 and higher*	3.14 [2.17; 4.54]	0.38 [0.22; 0.67]	3.14 [1.91; 5.16]	0.58 [0.29; 1.14]	2.91 [1.39; 6.08]	0.80 [0.29; 2.21]
Uses a wheelchair	2.59 [1.94; 3.45]	0.32 [0.19; 0.52]				
Uses a cane or crutches	2.14 [1.61; 2.84]	0.32 [0.20; 0.51]				
Uses a rollator	3.21 [2.23; 4.63]	0.41 [0.22; 0.78]				
Current disease-modifying treatments						
*Oral disease-modifying treatment*	Reference	Reference				
*Monoclonal disease-modifying treatment*	1.12 [0.81; 1.54]	0.57 [0.40; 0.81]				
*Injectable disease-modifying treatment*	0.70 [0.47; 1.06]	1.09 [0.77; 1.54]				
*Other disease-modifying treatment*	1.51 [0.68; 3.38]	0.47 [0.15; 1.44]				
*No disease-modifying treatment*	1.00 [0.75; 1.34]	0.83 [0.62; 1.11]				
Risk factors for MS onset and progression						
Reported MS cases in bloodline relatives	1.09 [0.78; 1.52]	1.05 [0.75; 1.46]				
Visual disturbances as first symptom	1.19 [0.93; 1.50]	1.01 [0.79; 1.28]				
Paresthesia as first symptom	1.00 [0.79; 1.27]	1.27 [1.00; 1.61]				
Gait problems as first symptom	1.17 [0.91; 1.49]	0.60 [0.46; 0.79]				
Spasms as first symptom	1.34 [0.91; 1.97]	0.52 [0.31; 0.87]				
Body Mass Index	1.01 [0.99; 1.03]	0.97 [0.95; 1.00]				
Smoking status						
*Unknown*	2.02 [0.53; 7.62]	0.74 [0.14; 3.85]				
*Never smoked*	Reference	Reference				
*Past smoker*	1.64 [1.22; 2.22]	0.79 [0.58; 1.09]				
*Current smoker*	1.23 [0.93; 1.61]	0.85 [0.65; 1.11]				
Self-reported comorbidities						
Mononucleosis	0.97 [0.69; 1.35]	1.07 [0.77; 1.48]				
High blood pressure	1.29 [0.64; 2.61]	0.97 [0.45; 2.09]				
Cancer	1.05 [0.75; 1.48]	0.87 [0.61; 1.25]				
Diabetes Type 1	2.08 [0.42; 10.36]	0.68 [0.07; 6.57]				
Diabetes Type 2	1.91 [0.80; 4.53]	1.31 [0.50; 3.39]				
Cardiovascular problems	1.07 [0.60; 1.91]	0.87 [0.47; 1.62]				

Model re-estimation including persons with a MSSS did not substantially alter the estimates of symptoms and SRDSS, except for pain and depression, which showed somewhat lower associations with the lowest VAS quartile. MSSS nominally also showed an association with the lowest VAS quartile (1.07 [0.99–1.15]). However, the 95% CI no longer suggested statistical significance at the 5% level.

The simultaneous quantile regression identified fewer variables to exhibit differential effects across the quantile strata (S3 Table). Only SRDSS, symptom load, and memory problems were retained from the main analysis. In addition, currently working was identified as a new influential factor.

### Discordant VAS and EQ-5D groupings

**Fig 2:**
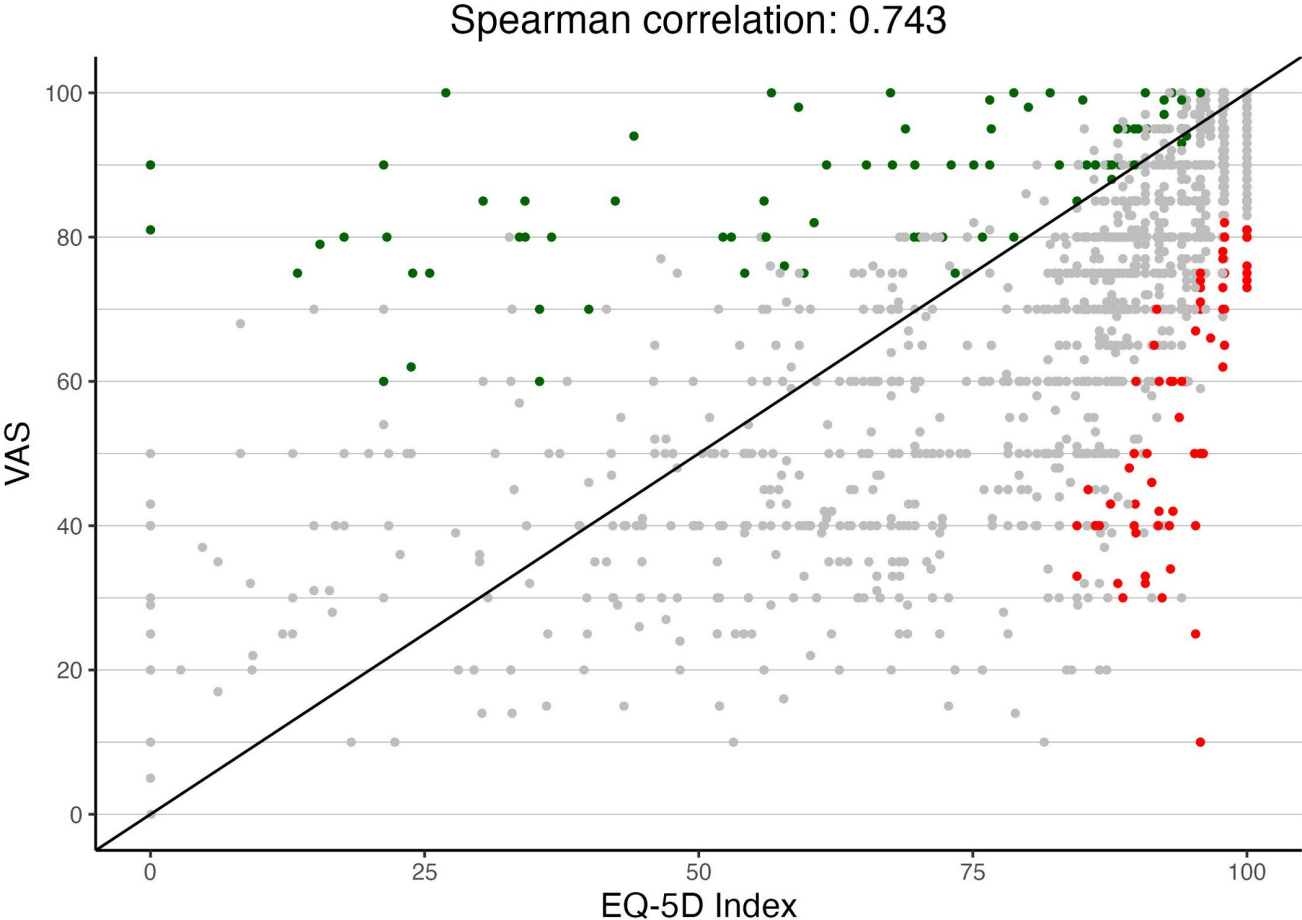
Correlation between VAS and EQ-5D and identification of discordant assessments. Green dots indicate individuals with low EQ-5D and high VAS. Red dots indicate individuals with high EQ-5D and low VAS.

The VAS and EQ-5D indicators were closely correlated (Spearman’s rho = 0.74). Nevertheless, VAS and EQ-5D measure somewhat different constructs, and we further examined discordant groupings (Fig 2 and Table 4). Green dots illustrate individuals with low EQ-5D-high VAS (n = 92, median EQ-5D 76.2, median VAS 90 point), red dots show individuals with high EQ-5D-low VAS (n = 82; median EQ-5D 95.8, median VAS 66.5 points). Descriptive analyses, which are shown in Table 3, suggest that a high ED-5D and low VAS was more frequent among younger PwMS who had a diagnosis for less long, and who reported a lower symptom burden when compared to PwMS with concordant VAS and EQ-5D assessments.

**Table 4:** Description of groups with concordant and discordant EQ-5D –VAS assessments.

	VAS score relative to EQ-5D index		
	lower VAS	within expectation	higher VAS
	N=82	N=1523	N=92
Sociodemographic characteristics			
Female sex	59 (72.0)	1122 (73.7)	60 (65.2)
Male sex	23 (28.0)	401 (26.3)	32 (34.8)
Age, median [IQR]	47 [36; 57]	49 [38; 57]	51 [40; 61.5]
Swiss citizen	68 (82.9)	1364 (89.6)	87 (94.6)
Currently employed	52 (63.4)	940 (61.7)	42 (45.7)
Disability benefits			
*Does not receive disability insurance*	2 (2.4)	101 (6.6)	3 (3.3)
*Has applied for disability insurance*	60 (73.2)	915 (60.1)	45 (48.9)
*Does receive disability insurance*	20 (24.4)	507 (33.3)	44 (47.8)
Education			
*Mandatory schooling completed*	5 (6.1)	57 (3.7)	3 (3.3)
*Apprenticeship*	15 (18.3)	634 (41.6)	39 (42.4)
*Highschool*	12 (14.6)	137 (9.0)	7 (7.6)
*Higher professional education*	14 (17.1)	214 (14.1)	20 (21.7)
*University degree*	33 (40.2)	413 (27.1)	20 (21.7)
*Other or unknown education status*	3 (3.7)	68 (4.5)	3 (3.3)
Living situation			
*Lives with family*	21 (25.6)	457 (30.0)	24 (26.1)
*Lives with friends/relatives*	3 (3.7)	37 (2.4)	4 (4.3)
*Lives alone*	20 (24.4)	329 (21.6)	23 (25.0)
*Lives with parents*	5 (6.1)	29 (1.9)	2 (2.2)
*Lives with spouse/partner*	32 (39.0)	628 (41.2)	38 (41.3)
*Other or unknown living situation*	1 (1.2)	43 (2.8)	1 (1.1)
Marital status			
*Divorced*	12 (14.6)	161 (10.6)	12 (13.0)
*Married*	33 (40.2)	775 (50.9)	44 (47.8)
*In registered partnership*	3 (3.7)	31 (2.0)	2 (2.2)
*Separated*	2 (2.4)	35 (2.3)	4 (4.3)
*Unmarried*	29 (35.4)	458 (30.1)	26 (28.3)
*Widowed*	2 (2.4)	39 (2.6)	4 (4.3)
*Unknown marital status*	1 (1.2)	24 (1.6)	0 (0.0)
MS disease characteristics			
Years since diagnosis, median [IQR]	7 [3; 18]	9 [4; 17]	12 [4; 20]
Age at diagnosis, median [IQR]	35 [27; 44]	37 [28; 45]	37 [28; 46]
Clinical MS phenotype			
*Clinically Isolated Syndrome (CIS)*	7 (8.5)	62 (4.1)	1 (1.1)
*Primary progressive MS (PPMS)*	4 (4.9)	158 (10.4)	18 (19.6)
*Relapsing-remitting MS (RRMS)*	58 (70.7)	1058 (69.5)	45 (48.9)
*Secondary progressive MS (SPMS)*	11 (13.4)	234 (15.4)	28 (30.4)
*Unclear clinical MS phenotype*	2 (2.4)	11 (0.7)	0 (0.0)
MS Severity Score, N, median [IQR]	42; 4.1 [1.9; 5.5]	849; 5.2 [2.1; 7.4]	46; 6.7 [4.5; 9.0]
Adjusted MS Severity Score, N, median [IQR]	42; 3.9 [1.6; 5.8]	849; 5.1 [2.3; 7.5]	46; 6.7 [4.9; 8.9]
Health-Related Quality of Life measurements			
EQ-5D index; median [IQR]	95.8 [91.9; 98.0]	90.7 [80.8; 96.2]	76.2 [52.8; 88.3]
Visual Analogue Scale; median [IQR]	66.5 [43; 75]	80 [60; 90]	90 [80; 95]
Current symptom burden			
Balance problems	21 (25.6)	549 (36.0)	36 (39.1)
Bladder problems	17 (20.7)	447 (29.3)	30 (32.6)
Depression	8 (9.8)	201 (13.2)	7 (7.6)
Fatigue	35 (42.7)	834 (54.8)	52 (56.5)
Gait problems	16 (19.5)	541 (35.5)	37 (40.2)
Gastrointestinal problems	9 (11.0)	359 (23.6)	20 (21.7)
Memory problems	17 (20.7)	340 (22.3)	14 (15.2)
Pain	13 (15.9)	465 (30.5)	26 (28.3)
Paresthesia	35 (42.7)	675 (44.3)	39 (42.4)
Spasms	11 (13.4)	453 (29.7)	31 (33.7)
Tremors	5 (6.1)	158 (10.4)	15 (16.3)
Muscle weakness	17 (20.7)	503 (33.0)	30 (32.6)
Number of MS Symptoms: median [IQR]	2.5 [0; 5]	4 [0; 8]	5 [1.8; 8]
*0–2 Symptoms*	41 (50.0)	608 (39.9)	27 (29.3)
*3–6 Symptoms*	28 (34.1)	409 (26.9)	33 (35.9)
*7 or more Symptoms*	13 (15.9)	506 (33.2)	32 (34.8)
Ambulatory impairments			
Self-reported disability status scale (SRDSS)			
*SRDSS 0–3*.*5*	72 (87.8)	1076 (70.7)	45 (48.9)
*SRDSS 4–6*.*5*	9 (11.0)	324 (21.3)	16 (17.4)
*SRDSS 7 and higher*	1 (1.2)	123 (8.1)	31 (33.7)
Uses a wheelchair	3 (3.7)	218 (14.3)	36 (39.1)
Uses a cane or crutches	7 (8.5)	248 (16.3)	16 (17.4)
Uses a rollator	2 (2.4)	133 (8.7)	11 (12.0)
Current disease-modifying treatments			
*Oral disease-modifying treatment*	32 (39.0)	513 (33.7)	26 (28.3)
*Monoclonal disease-modifying treatment*	10 (12.2)	302 (19.8)	22 (23.9)
*Injectable disease-modifying treatment*	16 (19.5)	213 (14.0)	6 (6.5)
*Other disease-modifying treatment*	1 (1.2)	28 (1.8)	1 (1.1)
*No disease-modifying treatment*	23 (28.0)	467 (30.7)	37 (40.2)
Risk factors for MS onset and progression			
Reported MS cases in bloodline relatives	9 (11.0)	222 (14.6)	8 (8.7)
Visual disturbances as first symptom	39 (47.6)	606 (39.8)	35 (38.0)
Paresthesia as first symptom	49 (59.8)	899 (59.0)	52 (56.5)
Gait problems as first symptom	16 (19.5)	477 (31.3)	33 (35.9)
Spasms as first symptom	8 (9.8)	125 (8.2)	9 (9.8)
Body Mass Index, N, median [IQR]	80; 23.7 [20.8; 26.6]	1472; 24.1 [21.4; 27.3]	89; 24.8 [21.7; 27.3]
Smoking status			
*Unknown*	0 (0.0)	10 (0.7)	1 (1.1)
*Never smoked*	40 (48.8)	657 (43.1)	41 (44.6)
*Past smoker*	18 (22.0)	326 (21.4)	20 (21.7)
*Current smoker*	24 (29.3)	530 (34.8)	30 (32.6)
Self-reported comorbidities			
Mononucleosis	13 (15.9)	225 (14.8)	8 (8.7)
High blood pressure	7 (8.5)	197 (12.9)	15 (16.3)
Cancer	2 (2.4)	39 (2.6)	3 (3.3)
Diabetes Type 1	0 (0.0)	6 (0.4)	1 (1.1)
Diabetes Type 2	0 (0.0)	24 (1.6)	4 (4.3)
Cardiovascular problems	3 (3.7)	57 (3.7)	8 (8.7)

The multivariable regression analysis (S4 Table) identified only a limited number of associations with AIC decreases of at least two points, namely the number of symptoms and SRDSS. The high EQ-5D–low VAS group was less likely to have a SRDSS of 7 or more (0.13 [0.02–0.97]) or 7 or more symptoms (0.49 [0.25–0.95]). The low EQ-5D–high VAS group was associated with an SRDSS of 7 or more (6.78 [3.74–12.28]).

## Discussion

Our analysis of 1679 PwMS illustrates the potential of distributional segmentation approaches to investigate reasons for low HRQoL. Removing the confounding effects of age and time since diagnosis, HRQoL was still predominantly associated with disease burden, characterized by gait impairment, total symptom load, or the presence of specific MS symptoms. Faster disease progression is an additional likely contributor to low HRQoL. Disease burden appeared to be more important than other characteristics, including sociodemographics, risk factors for MS progression, or comorbidities, which showed no associations in multivariable regression models.

Combined, our quantile-regression based segmentation with subsequent comparisons by HRQoL groups largely confirmed previous findings. By comparison, the sensitivity analysis based on simultaneous quantile regression identified fewer relevant factors and are more difficult to interpret for lay persons. Overall, the segmentation method offers a more intuitive approach to examine population- and health characteristics associated with low or high HRQoL. It also enables a simpler, direct comparison of two different HRQoL measures, as highlighted by our discordance analysis of EQ-5D and VAS indices.

Our main EQ-5D regression model suggests that this HRQoL measure correlates well with the EQ-5D subdomains of gait, pain, depression, and fatigue. However, the EQ-5D is generally not very responsive to small changes. By contrast, the VAS indicator does not focus on specific physical functions and has greater immediacy–VAS fluctuates on much smaller time scales and is influenced by a wide range of factors, including non-health-related conditions, such as weather [15]. It is, therefore, not entirely surprising that the VAS regression model generally exhibited weaker associations with disease burden while confirming the notable influences of depression, fatigue, pain, and muscle weakness on HRQoL. Additionally, our analysis of individual-level discordant VAS-EQ-5D assessments revealed interesting data pointing to the potential for further investigation. Notably, PwMS with a high ED-5D and low VAS tended to be younger, having a diagnosis for less long, and having a lower symptom burden than PwMS with concordant VAS and EQ-5D assessments. This finding points to a psychological component of well-being and ongoing coping and life-adjustment processes with MS [16]. This conclusion is corroborated by observations from the low EQ-5D–high VAS group, which tended to be more severely affected. EQ-5D tends to be relatively stable and less responsive to smaller changes over time or to changes in health-aspects that are not covered by the five domains. By contrast, VAS is a very general and subjective assessment of current HRQoL, that is influenced by a multitude of factors and exhibits much broader fluctuations. It has also been described previously that personal HRQoL assessments undergo baseline shifts as people grow older or increasingly cope with functional impairments [17]; that is, perceived general HRQoL as measured by VAS may increase or remain stable even though the structured, symptom-driven EQ-5D decreases due to increasing physical impairments. Combined, our observations suggest two conclusions: first, VAS assessments of persons with more severe, permanent physical impairments may undergo re-adjustments, with reference points for good HRQoL increasingly considering remaining physical functioning. Second, the analysis illustrates that low VAS assessments may not necessarily be tied to physical ailments, they can also be influenced by more immediate personal circumstances (e.g. stress) or mental well-being. Therefore, our method for identifying PwMS discordant EQ-5D–VAS self-assessments may help to identify persons in need of psychological or other support, but also highlights the need for closing gaps on health-related stressors not tied to MS symptoms or physical impairment.

Our findings generally align with existing literature on factors influencing HRQoL, including the detrimental effects of physical MS symptoms and impairment or disease progression. Contrary to other studies, however, our analysis did not indicate fatigue as a major independent contributor to low HRQoL. Other known factors, such as the role of comorbidities [4], could also not be corroborated by our study, possibly because of sample size limitations.

From a methodological viewpoint, our analysis illustrates the possible advantages of our distribution-based segmentation approach by showcasing its ease of interpretation and potential clinical applicability when compared to other quantile regression-based methods. MS management requires individuals with unexpectedly low HRQoL to be easily and reliably identified. Our method suggests a way forward but requires further optimization, for example, regarding optimal quantile boundaries. Unlike other studies investigating HRQoL trajectories, our analysis only included a cross-section. The quantile-based segmentation has the potential for longitudinal application, for example, by analyzing HRQoL change rates or repeated, intra-individual segment classifications.

Our findings also have implications for clinical practice, as factors associated with low HRQoL included symptoms like depression, fatigue, memory problems, muscle weakness, and pain, and high-level evidence supports non-pharmacological and pharmacological treatment options for symptomatic management of depression [18, 19], pain [20, 21], and fatigue [22] in MS. These “invisible” symptoms are quite frequent among persons with MS and exert a substantial burden on the lives of persons with MS [5]. They also pose significant job-related challenges and are associated with workforce drop-out [23]. Furthermore, because low HRQoL was found to be associated with depression, this indicator may also signal mental healthcare needs. Therefore, a more systematic HRQoL monitoring of health-related quality of life by means of the simple and low-effort EQ-5D may complement existing neurological assessments. Future studies should further examine the usefulness of monitoring for low HRQoL for routine care in longitudinal studies, for example, by investigating specific symptom burden changes associated with HRQoL decreases.

Our study is not without limitations. It should be emphasized that our study is unable to demonstrate (temporal) causality owing to its cross-sectional nature. Also, our study population was self-enrolled, and the majority of information is self-reported. Therefore, the effects of self-selection and reporting biases (e.g., social desirability biases) cannot be fully excluded. Furthermore, some factors (e.g., comorbidities) were only present in limited numbers, thus restricting the statistical power. Therefore, our findings require validation in different databases, as well as by including alternate measures of HRQoL, such as the 36-Item Short Form Survey (SF-36). Conversely, we consider the study population diversity, enabled by broad enrollment criteria, and the systematic approach of regression model building and co-variable specification as strengths. The distribution-based segmentation also requires no preconception of “good” or “bad” HRQoL while offering flexibility for further refinements. Furthermore, the method is easy to implement in any standard statistical software.

To summarize, our study found that low health-related quality of life was closely associated with physical impairment and specific MS symptoms, some of which are potentially mitigable. Depression, fatigue, memory problems, muscle weakness, and pain consistently emerged as symptoms with detrimental effects on HRQoL. Pharmacological and non-pharmacological MS symptom management, especially for depression, fatigue, pain, and muscle weakness, may warrant increased attention to preserve or improve HRQoL.

## Supporting information

S1 Fig Quantile-regression based HRQoL segmentation for the EQ-5D index. (DOCX)

S2 Fig Correlation between age and time since MS diagnosis. (DOCX)

S1 Table Selection of variables of interest and rationale for their inclusion. (DOCX)

S2 Table Sensitivity analysis–simultaneous quantile regression of EQ-5D. (DOCX)

S3 Table Sensitivity analysis–simultaneous quantile regression of VAS. (DOCX)

S4 Table Multivariable multinomial regression to identify associations with discordant EQ-5D - VAS assessments. (DOCX)
