# Supplementary figures and images for: Factors associated with low health-related quality of life in persons with multiple sclerosis: A quantile-based segmentation approach
Source: PLoS One. 2024 Nov 21;19(11):e0312486. doi: 10.1371/journal.pone.0312486 (PMC11581332; doi:10.1371/journal.pone.0312486)

**Supporting information**


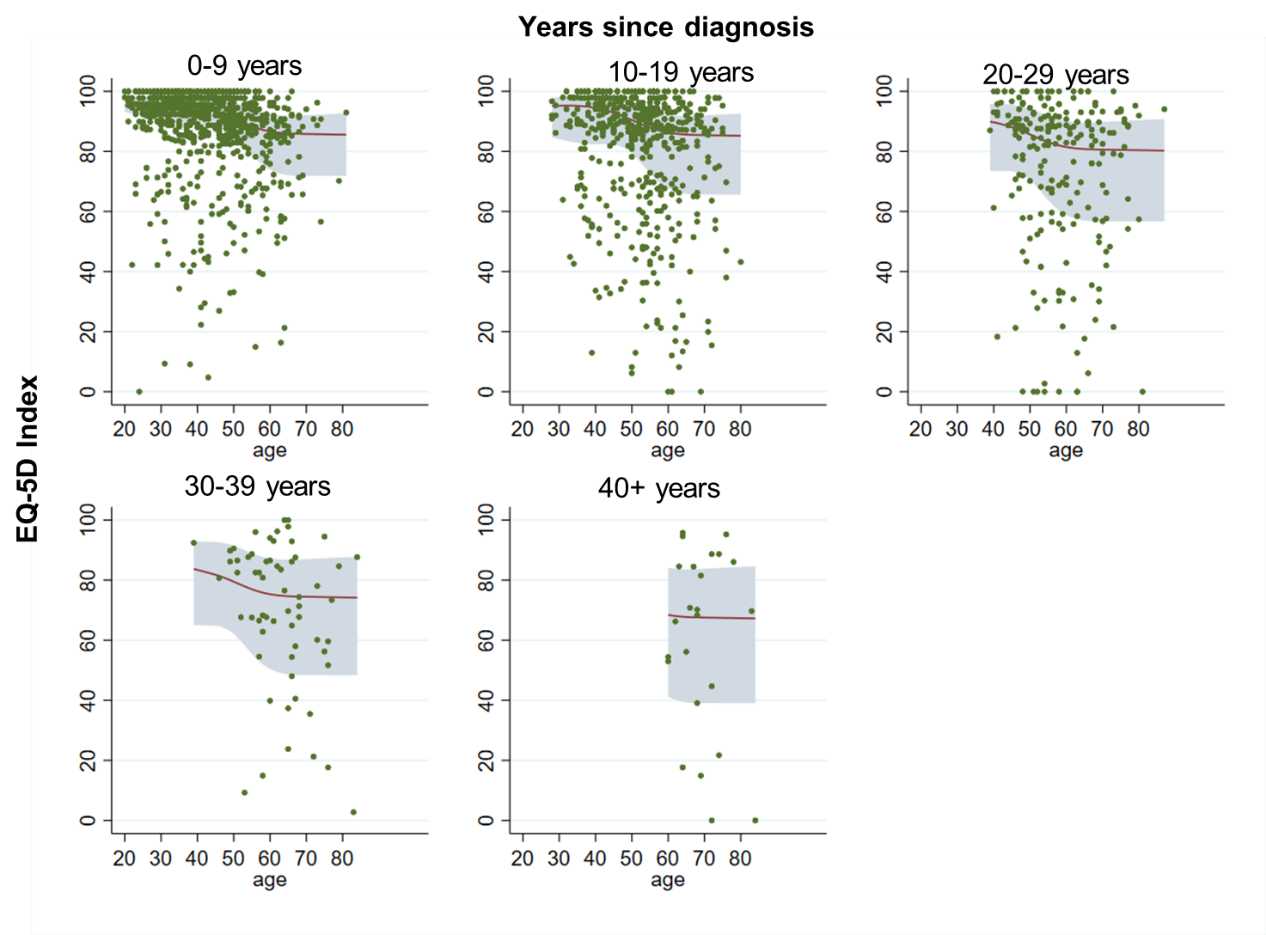


**S1 Fig**. **Quantile-regression based HRQoL segmentation for the EQ-5D index.**

Supplement: S1 Fig — (DOCX) [file pone.0312486.s001.docx]

**Supporting information**


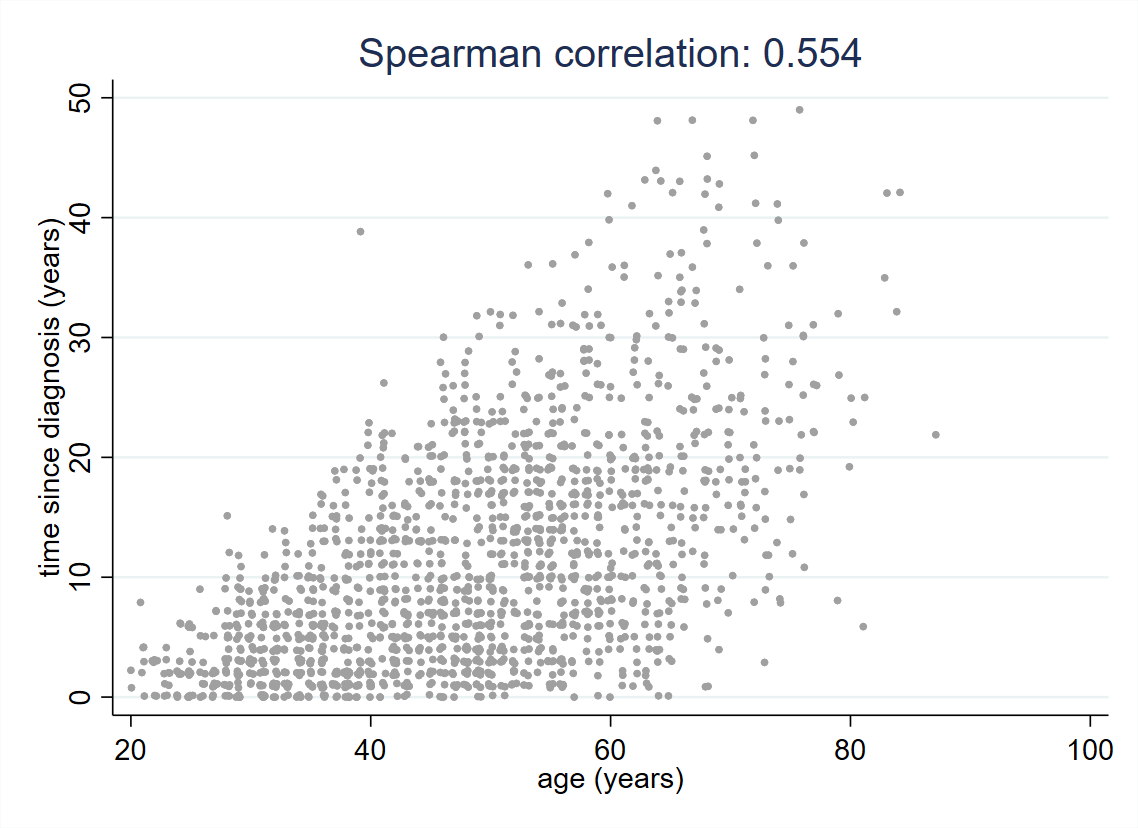
**S2 Fig. Correlation between age and time since MS diagnosis.**

Supplement: S2 Fig — (DOCX) [file pone.0312486.s002.docx]
