# Supplementary material for: Factors associated with low health-related quality of life in persons with multiple sclerosis: A quantile-based segmentation approach
Source: PLoS One. 2024 Nov 21;19(11):e0312486. doi: 10.1371/journal.pone.0312486 (PMC11581332; doi:10.1371/journal.pone.0312486)
Supplement: S1 Table — (DOCX) [file pone.0312486.s003.docx]

**Supporting information**

| **Variable** | **Rationale** | **References** |
| --- | --- | --- |
| **Pre-defined confounders** |  |  |
| Age* |  |  |
| Years since MS diagnosis* |  |  |
| Sex |  |  |
| Clinical MS phenotype |  |  |
| Self-reported disability status scale  (proxy for EDSS) | Indicator for gait impairment or social inclusion | Barin et al, MSARD, 2018; Mettler et al, MSARD, 2022 |
|  |  |  |
| **Exploratory variables** |  |  |
| Current disease-modifying treatments |  |  |
| No treatment | Possible health status indicator | Barin et al, MSARD, 2019 |
| Injectable treatments | Possible health status indicator; possibility of unwanted treatment effects | Barin et al, MSARD, 2019 |
| Oral treatments | Possible health status indicator; possibility of unwanted treatment effects | Barin et al, MSARD, 2019 |
| Infusion treatments | Possible health status indicator; possibility of unwanted treatment effects | Barin et al, MSARD, 2019 |
| Other treatments | Possible health status indicator; possibility of unwanted treatment effects | Barin et al, MSARD, 2019 |
|  |  |  |
| Sociodemographic characteristics |  |  |
| Swiss citizenship | Possible indicator for social inclusion |  |
| Education (mandatory, professional education, tertiary education) | Indicator for material wealth | Sanak et al, MSARD, 2023 |
| Partnership/Marital status | Possible indicator for social support | Mettler et al, MSARD, 2022 |
| Work status (full-time, part-time, retired,  not working) | Possible indicator for material wealth, health status or social inclusion | Sanak et al, MSARD, 2023;  Lehmann et al, Journal of Neurology, 2020 |
| Disability insurance benefits (receives benefits, applied for benefits, not receiving benefits) | Possible indicator for material wealth or health status | Sanak et al, MSARD, 2023 |
|  |  |  |
| Symptom burden |  |  |
| Number of self-reported MS symptoms | Possible indicator for MS severity with negative effects on HRQoL | Higginson et al, Journal of Palliative Care, 2006 |
| Balance as current symptom | Symptom with negative effects on HRQoL in prior study | Barin et al, MSARD, 2018 |
| Bladder as current symptom | Symptom with negative effects on HRQoL in prior study | Barin et al, MSARD, 2018; Wang et al, MSARD, 2018 |
| Depression as current symptom | Symptom with negative effects on HRQoL in prior study | Lobentanz et al, Acta Neurologica Scandinavica, 2004; Barin et al, MSARD, 2018 |
| Fatigue as current symptom | Symptom with negative effects on HRQoL in prior study | Lobentanz et al, Acta Neurologica Scandinavica, 2004; Barin et al, MSARD, 2018 |
| Gait as current symptom | Symptom with negative effects on HRQoL in prior study | Barin et al, MSARD, 2018 |
| Gastrointestinal tract as current symptom | Symptom with negative effects on HRQoL in prior study | Barin et al, MSARD, 2018 |
| Memory as current symptom | Symptom with negative effects on HRQoL in prior study | Barin et al, MSARD, 2018 |
| Pain as current symptom | Symptom with negative effects on HRQoL in prior study | Milinis et al, MSARD, 2016; Barin et al, MSARD, 2018 |
| Paresthesia as current symptom | Symptom with negative effects on HRQoL in prior study | Barin et al, MSARD, 2018 |
| Spasms as current symptom | Symptom with negative effects on HRQoL in prior study | Milinis et al, MSARD, 2016; Barin et al, MSARD, 2018 |
| Tremor as current symptom | Symptom with negative effects on HRQoL in prior study | Barin et al, MSARD, 2018 |
| Muscle weakness as current symptom | Symptom with negative effects on HRQoL in prior study | Barin et al, MSARD, 2018 |
|  |  |  |
| Self-reported comorbidities |  |  |
| High blood pressure | Comorbidities are known to reduce HRQoL | Barin et al, MSARD, 2018 |
| Cardiovascular diseases | Comorbidities are known to reduce HRQoL | Barin et al, MSARD, 2018 |
| Diabetes Type 1 | Comorbidities are known to reduce HRQoL | Barin et al, MSARD, 2018 |
| Diabetes Type 2 | Comorbidities are known to reduce HRQoL | Barin et al, MSARD, 2018 |
| Cancer | Comorbidities are known to reduce HRQoL | Barin et al, MSARD, 2018 |
|  |  |  |
| MS risk factors |  |  |
| Body Mass Index | Indicator for health status | Barin et al, MSARD, 2018 |
| Smoking status (current, ever, never) | Possible risk factor for faster MS progression | Hempel et al, MSJ, 2017; Briggs et al, MSJ, 2017 |
| Self-reported mononucleosis | Possible risk factor for faster MS progression | Endriz et al, Neurology Neuroimmunology and Neuroinflammation, 2017 |
| MS cases in bloodline family | Possible risk factor for MS progression; MS experience in close family | Hensiek et al, Neurology, 2007 |
|  |  |  |
| Risk factors associated with faster MS progression |  |  |
| Visual impairment as first symptom | Hallmark symptom for later lower MS symptom burden/ | Ajdacic-Gross et al, Frontiers Neurology, 2021 |
| Gait disturbance as first symptom | Hallmark symptom for later higher MS symptom burden/lower HRQoL | Ajdacic-Gross et al, Frontiers Neurology, 2021 |
| Spasms as first symptom | Hallmark symptom for later higher MS symptom burden/lower HRQoL | Ajdacic-Gross et al, Frontiers Neurology, 2021 |
| Paresthesia as first symptom | Hallmark symptom for later lower MS symptom burden/higher HRQoL | Ajdacic-Gross et al, Frontiers Neurology, 2021 |
| Multiple Sclerosis Severity Score | Indicator for MS impairment progression |  |

**S1 Table. Selection of variables of interest and rationale for their inclusion.**
