# Supplementary material for: Factors associated with low health-related quality of life in persons with multiple sclerosis: A quantile-based segmentation approach
Source: PLoS One. 2024 Nov 21;19(11):e0312486. doi: 10.1371/journal.pone.0312486 (PMC11581332; doi:10.1371/journal.pone.0312486)
Supplement: S2 Table — (DOCX) [file pone.0312486.s004.docx]

**Supporting information**

|  | **Replication of main analysis** | | | **De novo variable selection** | | |
| --- | --- | --- | --- | --- | --- | --- |
|  | **Q25** | **Median** | **Q75** | **Q25** | **Median** | **Q75** |
| Sociodemographic characteristics |  |  |  |  |  |  |
| Male sex | 0.70 [-0.72; 2.11] | 0.74 [-0.02; 1.50] | -0.11 [-0.57; 0.36] | 0.81 [-0.30; 1.93] | 0.60 [-0.33; 1.53] | 0.05 [-0.38; 0.47] |
| Currently employed | 0.40 [-1.36; 2.17] | 0.84 [-0.35; 2.03] | 0.06 [-0.56; 0.68] | 1.23 [-0.57; 3.03] | 0.43 [-0.73; 1.59] | 0.15 [-0.42; 0.72] |
| Disability benefits |  |  |  |  |  |  |
| Has applied for disability insurance | -4.24 [-7.39; -1.10] | -3.71 [-6.19; -1.23] | -2.49 [-3.98; -1.01] | -6.32 [-9.57; -3.06] | -3.19 [-6.14; -0.23] | -3.28 [-4.74; -1.83] |
| Does receive disability insurance | -5.35 [-7.37; -3.34] | -4.32 [-5.71; -2.93] | -2.01 [-3.18; -0.84] | -5.46 [-7.59; -3.32] | -4.78 [-6.10; -3.46] | -2.44 [-3.59; -1.30] |
| MS disease characteristics |  |  |  |  |  |  |
| Clinical MS phenotype |  |  |  |  |  |  |
| Clinically Isolated Syndrome (CIS) | -0.22 [-2.12; 1.68] | 0.66 [-0.59; 1.90] | 0.30 [-0.25; 0.85] | 0.38 [-1.73; 2.49] | 0.21 [-1.35; 1.77] | 0.31 [-0.16; 0.79] |
| Primary progressive MS (PPMS) | -1.85 [-4.44; 0.74] | -1.75 [-4.10; 0.61] | -2.02 [-3.37; -0.67] | -1.59 [-4.70; 1.52] | -1.28 [-3.74; 1.18] | -1.53 [-3.00; -0.07] |
| Secondary progressive MS (SPMS) | -2.57 [-6.01; 0.87] | -4.80 [-7.30; -2.30] | -2.97 [-4.58; -1.36] | -2.35 [-5.28; 0.59] | -4.03 [-6.54; -1.52] | -2.57 [-3.92; -1.22] |
| Unspecific phase | 1.64 [-5.30; 8.59] | 2.15 [-2.20; 6.50] | -3.56 [-5.68; -1.44] | -0.92 [-7.02; 5.18] | 0.42 [-5.07; 5.92] | -3.00 [-6.58; 0.59] |
| Current symptom burden |  |  |  |  |  |  |
| Depression | -11.99 [-16.90; -7.09] | -4.97 [-7.71; -2.24] | -2.51 [-3.71; -1.32] | -13.55 [-18.31; -8.78] | -5.57 [-8.39; -2.75] | -2.91 [-4.31; -1.51] |
| Fatigue | -0.94 [-2.37; 0.49] | -1.20 [-2.43; 0.03] | -1.05 [-2.00; -0.11] |  |  |  |
| Memory problems | -3.65 [-6.90; -0.39] | -0.61 [-2.25; 1.04] | -0.01 [-1.23; 1.21] | -3.06 [-6.23; 0.10] | -0.20 [-1.76; 1.35] | -0.41 [-1.70; 0.88] |
| Pain | -3.94 [-6.26; -1.62] | -2.52 [-4.27; -0.76] | -1.72 [-2.62; -0.83] |  |  |  |
| Muscle weakness | -2.53 [-4.96; -0.09] | -1.97 [-3.52; -0.43] | -1.14 [-2.19; -0.09] |  |  |  |
| Number of MS symptoms |  |  |  |  |  |  |
| 3-6 Symptoms | -1.51 [-3.18; 0.15] | -1.10 [-2.58; 0.37] | -1.13 [-2.19; -0.07] | -3.32 [-4.55; -2.10] | -2.94 [-3.82; -2.06] | -2.86 [-3.75; -1.97] |
| 7 or more Symptoms | -3.93 [-7.13; -0.73] | -4.27 [-7.00; -1.54] | -4.09 [-5.67; -2.50] | -8.15 [-10.64; -5.66] | -8.42 [-10.14; -6.70] | -6.73 [-8.04; -5.41] |
| Ambulatory impairments |  |  |  |  |  |  |
| Self-reported disability status scale (SRDSS) |  |  |  |  |  |  |
| SRDSS 4-6.5 | -15.66 [-19.61; -11.72] | -8.37 [-11.13; -5.61] | -4.70 [-5.84; -3.57] | -15.22 [-19.24; -11.19] | -7.48 [-10.60; -4.36] | -4.29 [-5.41; -3.17] |
| SRDSS 7 and higher | -53.56 [-60.80; -46.32] | -37.32 [-43.43; -31.20] | -26.76 [-33.40; -20.11] | -54.50 [-60.89; -48.10] | -38.66 [-45.13; -32.20] | -27.08 [-33.93; -20.22] |
| Current disease-modifying treatments |  |  |  |  |  |  |
| Monoclonal disease-modifying treatment | -0.82 [-2.41; 0.77] | -0.69 [-1.79; 0.41] | -0.25 [-0.95; 0.46] |  |  |  |
| Injectable disease-modifying treatment | 0.44 [-1.04; 1.92] | 0.15 [-0.78; 1.07] | 0.09 [-0.42; 0.60] |  |  |  |
| Other disease-modifying treatment | -1.67 [-9.69; 6.35] | 1.53 [-5.35; 8.41] | -0.07 [-3.22; 3.08] |  |  |  |
| No disease-modifying treatment | -1.42 [-2.82; -0.02] | -0.17 [-1.14; 0.80] | -0.02 [-0.47; 0.44] |  |  |  |
| Risk factors for MS onset and progression |  |  |  |  |  |  |
| Gait problems as first symptom |  |  |  | -2.47 [-3.86; -1.08] | -0.73 [-1.74; 0.27] | -0.72 [-1.58; 0.14] |
| Spasms as first symptom |  |  |  | -1.75 [-4.64; 1.13] | -1.24 [-2.97; 0.48] | -0.32 [-1.78; 1.15] |
| Body Mass Index | -0.14 [-0.25; -0.03] | -0.14 [-0.23; -0.04] | -0.07 [-0.13; -0.01] | -0.15 [-0.25; -0.06] | -0.13 [-0.22; -0.04] | -0.09 [-0.16; -0.01] |
| Smoking status |  |  |  |  |  |  |
| Unknown | 2.41 [-4.45; 9.27] | 1.04 [-3.71; 5.78] | -0.02 [-4.00; 3.96] |  |  |  |
| Past smoker | -1.30 [-2.79; 0.19] | -1.33 [-2.25; -0.40] | -0.60 [-1.27; 0.08] |  |  |  |
| Current smoker | -0.62 [-1.94; 0.71] | -0.64 [-1.51; 0.23] | -0.12 [-0.59; 0.35] |  |  |  |

**S2 Table. Sensitivity analysis – simultaneous quantile regression of EQ-5D.**
