# Supplementary material for: Factors associated with low health-related quality of life in persons with multiple sclerosis: A quantile-based segmentation approach
Source: PLoS One. 2024 Nov 21;19(11):e0312486. doi: 10.1371/journal.pone.0312486 (PMC11581332; doi:10.1371/journal.pone.0312486)
Supplement: S3 Table — (DOCX) [file pone.0312486.s005.docx]

**Supporting information**

|  | **Replication of main analysis** | | | **De novo variable selection** | | |
| --- | --- | --- | --- | --- | --- | --- |
|  | **Q25** | **Median** | **Q75** | **Q25** | **Median** | **Q75** |
| Sociodemographic characteristics |  |  |  |  |  |  |
| Male sex | 0.60 [-1.98; 3.18] | -0.14 [-1.98; 1.71] | 0.82 [-0.96; 2.59] | 0.85 [-1.78; 3.48] | 0.25 [-1.64; 2.15] | 1.18 [-0.91; 3.27] |
| Other citizenship | 3.77 [0.05; 7.48] | 2.08 [-0.45; 4.62] | 3.32 [0.98; 5.66] |  |  |  |
| Currently employed |  |  |  | 3.58 [0.32; 6.84] | 2.09 [-0.54; 4.73] | -1.31 [-3.54; 0.91] |
| Disability benefits |  |  |  |  |  |  |
| Has applied for disability insurance | -17.88 [-23.87; -11.90] | -13.63 [-20.60; -6.67] | -11.28 [-15.68; -6.87] | -21.60 [-26.65; -16.54] | -14.39 [-22.26; -6.53] | -10.32 [-15.09; -5.56] |
| Does receive disability insurance | -11.59 [-15.20; -7.98] | -8.02 [-10.49; -5.54] | -5.59 [-8.25; -2.93] | -12.32 [-16.12; -8.52] | -8.56 [-11.21; -5.91] | -5.77 [-8.38; -3.16] |
|  |  |  |  |  |  |  |
| MS disease characteristics |  |  |  |  |  |  |
| Clinical MS phenotype |  |  |  |  |  |  |
| Clinically Isolated Syndrome (CIS) | -1.68 [-6.89; 3.54] | -1.88 [-5.61; 1.86] | -0.21 [-3.64; 3.22] | -0.52 [-6.88; 5.83] | -0.75 [-3.84; 2.34] | -0.66 [-3.62; 2.31] |
| Primary progressive MS (PPMS) | -3.29 [-8.37; 1.79] | -2.69 [-6.58; 1.21] | -2.90 [-6.39; 0.59] | -4.18 [-9.26; 0.90] | -1.26 [-5.23; 2.71] | -1.78 [-5.05; 1.49] |
| Secondary progressive MS (SPMS) | -6.70 [-11.17; -2.24] | -1.50 [-5.26; 2.27] | -2.07 [-5.36; 1.21] | -6.49 [-11.88; -1.09] | -0.82 [-4.46; 2.82] | -2.08 [-6.38; 2.21] |
| Unspecific phase | 10.13 [-17.20; 37.47] | 0.05 [-12.13; 12.24] | 3.39 [-9.07; 15.86] | 8.48 [-18.51; 35.47] | 1.89 [-11.88; 15.66] | 2.10 [-10.50; 14.70] |
| Ambulatory impairments |  |  |  |  |  |  |
| Self-reported disability status scale (SRDSS) |  |  |  |  |  |  |
| *SRDSS 4-6.5* | -13.86 [-18.04; -9.68] | -13.43 [-16.80; -10.06] | -8.04 [-11.02; -5.05] | -13.27 [-17.59; -8.96] | -12.40 [-16.37; -8.44] | -8.21 [-11.16; -5.25] |
| *SRDSS 7 and higher* | -22.33 [-28.71; -15.96] | -17.68 [-23.38; -11.97] | -10.78 [-16.03; -5.54] | -20.64 [-27.15; -14.12] | -16.73 [-22.67; -10.79] | -10.07 [-15.54; -4.60] |
|  |  |  |  |  |  |  |
| Current symptom burden |  |  |  |  |  |  |
| Depression | -6.75 [-12.26; -1.24] | -4.41 [-8.67; -0.15] | -4.34 [-7.78; -0.89] |  |  |  |
| Fatigue | -3.24 [-6.61; 0.12] | -0.95 [-3.73; 1.82] | -2.23 [-4.46; 0.00] |  |  |  |
| Memory problems | -6.00 [-10.06; -1.94] | -5.01 [-8.13; -1.89] | -1.13 [-4.18; 1.91] | -7.38 [-11.48; -3.28] | -4.05 [-7.25; -0.86] | -0.74 [-4.16; 2.68] |
| Pain | -4.56 [-8.47; -0.65] | -3.98 [-6.50; -1.45] | -4.09 [-6.83; -1.34] |  |  |  |
| Paresthesia | -2.24 [-4.87; 0.38] | -2.56 [-4.69; -0.43] | -2.02 [-3.73; -0.32] |  |  |  |
| Muscle weakness | -2.47 [-6.16; 1.21] | -3.76 [-6.75; -0.78] | -2.97 [-5.50; -0.43] |  |  |  |
| Number of MS symptoms |  |  |  |  |  |  |
| *3-6 Symptoms* | -2.46 [-6.15; 1.22] | -3.52 [-6.72; -0.32] | -1.12 [-3.85; 1.62] | -5.74 [-8.86; -2.62] | -7.72 [-10.10; -5.34] | -5.59 [-7.38; -3.81] |
| *7 or more Symptoms* | -6.74 [-12.20; -1.28] | -6.08 [-10.75; -1.40] | -3.92 [-8.41; 0.58] | -14.51 [-19.32; -9.70] | -15.25 [-18.59; -11.91] | -14.01 [-17.24; -10.78] |

**S3 Table.** **Sensitivity analysis – simultaneous quantile regression of VAS.**
