# Supplementary material for: Factors associated with low health-related quality of life in persons with multiple sclerosis: A quantile-based segmentation approach
Source: PLoS One. 2024 Nov 21;19(11):e0312486. doi: 10.1371/journal.pone.0312486 (PMC11581332; doi:10.1371/journal.pone.0312486)
Supplement: S4 Table — (DOCX) [file pone.0312486.s006.docx]

**Supporting information**

|  | **Multivariable, N = 1697** | |
| --- | --- | --- |
|  | **high EQ-5D low VAS** | **low EQ-5D high VAS** |
|  | **RRR [95% CI]** | **RRR [95% CI]** |
|  | **N=82** | **N=92** |
| Sociodemographic characteristics |  |  |
| Age | 1.01 [0.99; 1.03] | 0.99 [0.97; 1.01] |
| Female sex | Reference | Reference |
| Male sex | 1.23 [0.74; 2.05] | 1.35 [0.85; 2.15] |
| Ambulatory impairments |  |  |
| Self-reported disability status scale (SRDSS) |  |  |
| *SRDSS 0-3.5* | Reference | Reference |
| *SRDSS 4-6.5* | 0.46 [0.21; 0.98] | 1.29 [0.67; 2.47] |
| *SRDSS 7 and higher* | 0.13 [0.02; 0.97] | 6.78 [3.74; 12.28] |
| Current symptom burden |  |  |
| Number of MS Symptoms |  |  |
| *0-2 Symptoms* | Reference | Reference |
| *3-6 Symptoms* | 1.11 [0.67; 1.85] | 1.60 [0.92; 2.76] |
| *7 or more Symptoms* | 0.49 [0.25; 0.95] | 0.97 [0.54; 1.73] |

**S4 Table.** **Multivariable multinomial regression to identify associations with discordant EQ-5D - VAS assessments.**
